# The potential of copper oxide nanoparticles in nanomedicine: A comprehensive review

**DOI:** 10.1016/j.biotno.2024.06.001

**Published:** 2024-06-05

**Authors:** Mahalakshmi Devaraji, Punniyakoti V. Thanikachalam, Karthikeyan Elumalai

**Affiliations:** Department of Pharmaceutical Chemistry, Saveetha College of Pharmacy, Saveetha Institute of Medical and Technical Sciences, Chennai, India

**Keywords:** Copper oxide nanoparticles, Antimicrobial activity, Anticancer, Wound healing, Plant growth, In vitro and in vivo toxicity

## Abstract

Nanotechnology is a modern scientific discipline that uses nanoparticles of metals like copper, silver, gold, platinum, and zinc for various applications. Copper oxide nanoparticles (CuONPs) are effective in biomedical settings, such as killing bacteria, speeding up reactions, stopping cancer cells, and coating surfaces. These inorganic nanostructures have a longer shelf life than their organic counterparts and are chemically inert and thermally stable. However, commercial synthesis of NPs often involves harmful byproducts and hazardous chemicals. Green synthesis for CuONPs offers numerous benefits, including being clean, harmless, economical, and environmentally friendly. Using naturally occurring organisms like bacteria, yeast, fungi, algae, and plants can make CuONPs more environmentally friendly. CuONPs are expected to be used in nanomedicine due to their potent antimicrobial properties and disinfecting agents for infectious diseases. This comprehensive review looks to evaluate research articles published in the last ten years that investigate the antioxidant, anticancer, antibacterial, wound healing, dental application and catalytic properties of copper nanoparticles generated using biological processes. Utilising the scientific approach of large-scale data analytics. However, their toxic effects on vertebrates and invertebrates raise concerns about their use for diagnostic and therapeutic purposes. Therefore, biocompatibility and non-toxicity are crucial for selecting nanoparticles for clinical research.

## Abbreviations

**Short name-full name**
CuONPs: Copper oxide nanoparticles
NPs: Nanoparticles
FTIR: Fourier Transform Infrared Spectroscopy
XRD: X-Ray Diffraction
UV: Ultra Violet spectroscopy
TEM: Transmission Electron Microscopy
SEM: Scanning Electron Microscopy
DLS: Dynamic Light Scattering
EDX: Energy Dispersive X-Ray
FESEM: Field Emission Scanning Electron Microscopy
HA: Haemagglutination
NDV: Newcastle Disease Virus
MIC: Minimum Inhibitory Concentration
FRAP: Ferrous Reduced Antioxidant Power
FTC: Ferric Thiocyanate
TPC: Total Phenolic Content
SOD: Superoxide Dismutase
GST: Glutathione S-Transferase
GP: Glutathione Peroxidase
GR: Glutathione Reductase
ZnONPs: Zinc Oxide Nanoparticles
AgONPs: Silver Oxide Nanoparticles

## 1 Introduction

Nanotechnology is a contemporary scientific discipline in the 21st century. Nanoparticles, which fall within the 1–100 nm nanoscale range, exhibit unique characteristics in terms of their structures and chemical, physical, and mechanical properties compared to their larger forms.^1^ Utilising nanoparticles of metals such as copper, silver, gold, platinum, and zinc has been a common practice in medicine for some time.^2^, ^3^, ^4^, ^5^ Transitional metal oxides have proven functional in various fields, such as catalytic processes, medicine, dental care, biosensors, energy, agriculture and food production, technology, beauty products, and environmental protection.^6^^,^^7^ Copper oxide nanoparticles (CuONPs) have interesting properties in several biomedical settings, such as being highly effective at killing bacteria, speeding up reactions, stopping cancer cells from growing, and coating surfaces.^8^ Natural CuONPs are inexpensive and plentiful. Different oxidation states, such as Cu0, CuI, CuII, and CuIII, contain these NPs,^9^ exhibiting intriguing physicochemical properties. They are chemically inert and thermally stable,^10^ and their ∼2 eV band gap energy has given them fascinating significance.

Being inorganic, CuONPs have a longer shelf life than their organic counterparts. These nanostructures are monoclinic and p-type semiconductors. Obtaining NPs with the required size and morphology relies heavily on the ability of synthetic methods to control them. The commercial synthesis of NPs typically employs physical and chemical processes. Various deposition methods, such as chemical vapour deposition,^11^, ^12^, ^13^ electrochemical techniques,^14^ sol-gel synthesis,^15^^,^^16^ precipitation,^17^ hydrothermal processes,^18^^,^^19^ chemical bath deposition,^20^ sonochemical route,^21^ and chemical reduction,^22^ involve the use of powerful reducing agents, solvents that are organic, and hazardous chemicals, resulting in the generation of harmful byproducts. Potent synthetic compounds like hypophosphite,^23^ sodium borohydride,^24^ and hydrazine,^25^ used as reducing agents in the chemical process, increase the toxicity of produced nanoparticles. Nevertheless, in contrast to green production, physical processes such as gamma rays, pulsed lasers, and mechanical milling demand significant energy. They are more time-consuming to expand on a larger scale.^26^^,^^27^ A substantial focus has been on developing an environmentally friendly approach to producing metal NPs. Choosing green synthesis for Cu and CuONPs offers numerous benefits, including being clean, harmless, economical, and environmentally friendly compared to traditional chemical and physical methods. Instead of employing harmful and expensive chemicals, it makes use of naturally occurring organisms, including bacteria,^28^ yeast,^29^ fungi,^30^ algae,^31^ and plants.^32^ Because they can act as a reduction agent, a stabilising agent, and a capping agent all at the same time, plants that are high in bioactive compounds are the best choice for making CuONPs in an environmentally friendly way.

The utilisation of CuONPs is expected in nanomedicine due to their potent antibacterial properties and disinfecting agents for infectious diseases. These substances are used in wound dressings due to their bactericidal properties against gram-positive and gram-negative bacteria.^33^ CuONPs have fungicidal effects against specific fungus strains. We employ CuONPs as dopamine, glucose, lipid levels, lactate, and DNA biosensors. Their significance as possible anticancer agents for breast, lung, prostate gland, kidney, and glioblastoma cancer is paramount. In addition, they function as nanocarriers.^34^^,^^35^ It plays a crucial role in cellular respiration, the regulation of neurotransmitters, the formation of collagen proteins, and the metabolism of food, including the iron required for proteins and enzymes.^10^

Although CuONPs have potential in biomedicine, their toxic effects on vertebrates and invertebrates raise concerns about their usage for diagnostic and therapeutic reasons. CuO NPs can cause oxidative stress in living cells by over-generating reactive oxygen species (ROS), causing DNA and organelle damage. Size, surface charge, and dissolution are the leading causes of CuONP toxicity in vitro and in vivo.^36^ Thus, biocompatibility and non-toxicity are crucial for selecting nanoparticles for clinical research.

This review explains CuONPs to readers in-depth, including how they are made, their (exclusively) biological applications, and the results of a toxicological study.

## 2 Synthesis techniques for copper oxide nanoparticles

**Fig. 1:**
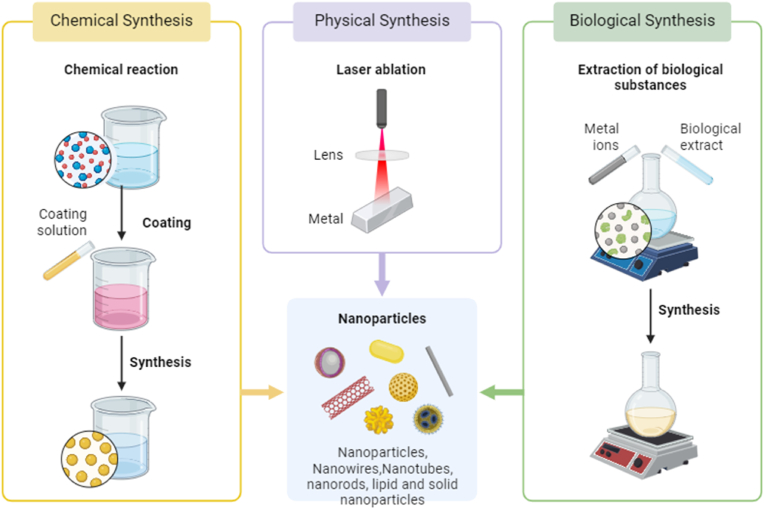
Synthesis techniques for copper oxide nanoparticles.

Preparing metal nanoparticles in a specific way reduces particle size and stabilises the nanoparticle. Thus, it's essential to use an appropriate approach. Copper nanomaterials are attracting much interest since they are abundant, readily available, and relatively inexpensive compared to precious metals like gold and silver. There are three primary methods employed to generate CuONPs: physical, chemical, and biological (Fig. 1).

### 2.1 Physical process

Physical methods like laser ablation, mechanical milling, and sputtering typically produce nanoparticles in large quantities. This category consists solely of synthetic, top-down techniques. This technique destroys material to create NPs by breaking it into smaller pieces. We developed CuONPs via the physical synthesis method using laser ablation.

In this technique, laser irradiation induces the decomposition of the precursor into NPs. CuONPs with an 8–10 nm diameter were formed when a laser source (Nd-YAG) was applied to Cu metal for 10 min.

The pulse parameters included a width of 7 ns, a wavelength of 1064 nm, and a frequency of 5 Hz.^37^ Using an ultrasonic-assisted grinding technique was the most straightforward mechanical approach to creating CuONPs. We set the grinding speed at 256 revolutions per minute and used cupric acetate as the precursor. Finally, researchers successfully produced CuONPs measuring 20 nm.^38^ Researchers used iron metal as a reducing agent and covellite (CuS) and chalcocite (Cu2S) as precursors to make CuONPs with average diameters of 16 nm, which changed over time and based on their size.^39^ Researchers have also noted that sputtering is a physical route to creating CuO NPs. We anneal thin layers of NPs to achieve this effect.^40^ We synthesised nanoparticles of varying sizes, starting with Cu as the initial material and solutions of different concentrations. The physical method's benefits include the generation of high-purity, uniformly sized CuONPs.^41^ The physical technique of synthesising CuONPs has clear benefits but has significant drawbacks, such as high cost, complicated operation, and excessive power and energy consumption.

### 2.2 Chemical process

All chemical synthesis techniques use a bottom-up process in which smaller building blocks assemble into larger ones to generate NPs. These strategies are efficient because they create products of consistent size and shape at low cost and don't require expensive, high-throughput machinery.

For the chemical synthesis of CuO NPs, sol-gel is the method of choice due to its simplicity, scalability, and low cost. The conversion of copper carbonate species at an acidic pH of 5.8 and a calcination temperature over 250 °C resulted in the production of CuONPs. These nanoparticles had controlled surface spaces and crystalline diameters ranging from 100 to 140 nm.^42^ The combination of Lantana camara extract, copper chloride, and sodium hydroxide produced nanoparticles with a particle size of 17 nm. These nanoparticles demonstrated photocatalytic activity.^43^ This finding highlights the potential of the Sol-gel approach for the environmentally friendly synthesis of nanoparticles. Generating stable nanoparticles using an easy process, thermal breakdown in liquid phase, has garnered interest because of its antibacterial efficacy against *Escherichia coli*.^44^^,^^45^ The synthesis of CuO NPs via co-precipitation is a straightforward and effective process. Phiwdang et al. (2013) used a precipitation procedure to create CuO NPs of varying sizes and shapes from two distinct precursors: copper nitrate and copper chloride.^46^ In another investigation, CuO NPs were produced using copper sulphate, copper chloride and copper as starting materials. This technique produced CuONPs of various shapes and sizes.^47^ When manufacturing different nanoparticles, capping agents are commonly used to stabilise and reduce agents. These agents, along with the precursors, are usually added at the start of the reaction.^48^ Using copper sulphate as its precursor and aniline as an encompassing agent, researchers created CuO NPs in a study.^49^ The sonochemical approach creates NPs by subjecting them to ultrasonic vibrations. A recent experiment produced Cetyltrimethylammonium bromide-stabilised CuO NPs from a copper sulphate precursor.^50^ The chemical method used to synthesise CuONPs and other transition metallic oxide NPs has notable limitations, including high energy consumption, contamination of the environment, and expensive and toxic chemicals, as reported in multiple studies.^51^^,^^52^

### 2.3 Copper nanoparticles are synthesised using a green approach

**Fig. 2:**
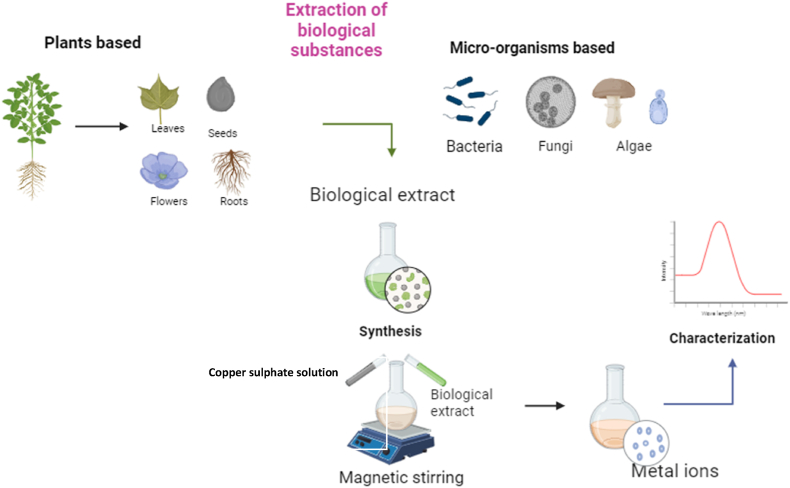
Green synthesis of copper oxide nanoparticles.

“Green biosynthesis” or “biological fabrication” of NPs is a method that incorporates plant and microbial extracts, including those from bacteria, fungi, algae, and actinomycetes (Fig. 2). Biogenic manufacturing is a simple, inexpensive, and environmentally friendly alternative to physical and chemical processes that produce NPs that are biocompatible and non-toxic. These alternatives avoid using costly and potentially harmful chemicals such as solvents and stabilising agents.^53^ The molecules produced by this synthesis process are more stable, and they are also easy to repeat, cheap, reliable, sustainable, and have less waste. Green synthesis does not necessitate capping moieties because microbial enzymes serve as both capping and reducing agents. This leads to stabilised nanoparticles and secondary metabolism products in plant extracts, including phenol compounds, flavonoids, alkaloids, and terpenoids.

#### 2.3.1 Copper oxide nanoparticles synthesised by plants

Plant extracts have been widely used to create CuONPs.^54^, ^55^, ^56^ The time-consuming culture and continual sterile conditions needed for nanoparticle creation by bacteria, algae, or fungi are significant drawbacks compared to plant-mediated nanoparticle synthesis. See Refs. 57,58 Fabrication from plants has many advantages, including being a straightforward procedure, being safe, using less energy, and improving the stability of nanoparticles. This method involves combining plant extracts with metal salts and letting the reaction proceed at room temperature for one to 3 h. Nanoparticles containing bioactive metabolites such as flavonoids, protein molecules, tannins, terpenoids, and phenolic compounds were created when plant extracts transformed metallic salts.^59^, ^60^, ^61^, ^62^, ^63^, ^64^ Copper salts are reduced by the plant extract, which produces electrons. The phytochemicals undergo a reduction reaction with the copper ion and then transform into CuONPs.^65^, ^66^, ^67^, ^68^, ^69^ CuONPs have been manufactured using many different kinds of plants, as shown in Table 1.

**Table 1:** Copper oxide nanoparticles synthesised by plants.

Plant name	Parts used	Phytochemical constituents involved in bio-reduction	Shape	Size (nm)	Reference
Phaseolus vulgaris	Fruit	phytic acids, Phenolic compounds, saponins and protease Inhibitors	Spherical	26.6	70
Murraya koenigii	Leaves	Phenols and flavonoids	Spherical	12	71
Manilkara zapota	Leaves	Flavonoids, Glycosides, Triterpenoids, And Polyphenol	Spherical	18.9–45.2	72
Camellia sinensis	Leaves	Polyphenols	Spherical	67–99	73
Eucalyptus globulus	Leaves	Flavonoids, Phenol, triterpenoids, and tannins	Spherical, Oval-shaped and Cuboidal	12–68	74
Lawsonia inermis	Leaves	Mannitol, Hennotannic acid, and alkaloids	Spherical	22–38	75
Terminalia bellirica	Fruits	Tannins	Spherical	9–14	76
Ailanthus altissima	Leaves	Proteins, phenols, and Alkenes	Spherical	5–20	77
Azadirachta indica	Leaves	Phenols and flavonoids	Spherical	12	71
Beta vulgaris	Leaves	phenol and Alcohol	Spherical And Irregular	11–63	78
Eclipta prostrate	Leaves	Steroids, triterpenes, and Flavonoids,	Spherical	23–57	79
Nilgirianthus ciliates	Leaves	Sapanonin, Phenol, and Tannins	Spherical	20	80
Olea europaea	Leaves	Flavonoids	Spherical	20–50	81
Cissus arnottiana	Leaves	Biomolecules	Spherical	60–90	82
Ficus religiosa	Leaves	Alkaloids, flavonoids, and Terpenoids	Spherical	577	83
Hibiscus	Leaves	Phenols and Flavonoids	Spherical	12	71
Moringa oleifera	Leaves	Phenols and Flavonoids	Spherical	12	71
Saccharum Officinarum	Stem	Glucose, sucrose, and Fructose	Spherical, Square, cube, Plate, Rectangular	29.5–60.5	84
*Tamarindus indica*	Leaves	Phenols and Flavonoids	Spherical	12	71
Tridax procumbens	Leaves	Hexadecen, pentadecne, And squalene	Spherical	16	85

#### 2.3.2 Copper oxide nanoparticles synthesised by fungai

Recently, several different fungal species have discovered applications in the manufacturing of nanoparticles from metals, including copper oxide products. Fungi have various advantages over other microbes when it comes to nanoparticle manufacturing. Unlike bacteria, fungi can withstand the conditions in a bioreactor or other growth chamber, including agitation, low pressure, and others. Nanoparticles can be biofabricated by using cell-free microbial extracts as encapsulating, reducing, or catalytic agents.^86^ Trichoderma species, a famous mould, do not contribute to creating nanoparticles made of copper oxide. Still, they make many valuable substances, including diketopiperazine, digestive enzymes, polyketides, terpenes, glycolipids, pyrones, and more.^87^^,^^88^ Fungi create nanoparticles through two main mechanisms: intracellular and extracellular pathways.

Nanoparticles produced by fungi may be smaller, more dispersed, and have better dimensions than those made by other means, such as the extracellular route.^89^ The extracellular approach offers several advantages when creating nanoparticles. The generated nanoparticles may not have any biological constituents. The use of fungi as stabilising and reducing agents in the production of nanoparticles has predominantly been exploited for this purpose due to the release of various compounds by fungi.^90^ CuONPs and other metal oxide nanoparticles have been synthesised using fungal strains. Table 2 displays the fungal production of copper oxide nanoparticles.

**Table 2:** Copper oxide nanoparticles synthesised by Fungai.

Fungai culture	Precursor	Size (nm)	Shape	Characterisation techniques	Reference
Aspergillus oryzae Cohn	Copper Sulphate	15.8	Spherical	UV–Vis, SEM, TEM and XRD	91
Aspergillus fumigatus Fresen.	Copper nitrate	8	Spherical	UV–Vis, FTIR, EDX, HRTEM, Zeta, XRD,	92
Aspergillus flavus Link	Copper Sulphate	20	Spherical	UV–Vis, XRD, TEM, FTIR	93
Aspergillus terreus Cohn	Copper Sulphate	15.8	Spherical	TEM, SEM UV–Vis, FT-IR, and XRD	94
Fusarium oxysporum Schltdl.	Metallic copper	93–115	**-**	UV–Vis TEM, AND SEM	95
Botrytis cinerea Pers.	Copper Sulphate	60–80	Spherical	TEM, XRF, SDD and EDX,	96
Hypocrea lixii Pat.	Metallic copper	24.5	Spherical	UV–Vis TEM and SEM	97
Stereum hirsutum Pers.	Copper salts	4–5	monodispersed, spherical	FTIR, TEM, Zeta Potential, and XRD	98
Trichoderma harzianum Rifai	Copper Sulphate	5–18	Dense agglomerate and spherical	UV–Vis, XRD TEM, SEM and DLS,	99
Neurospora crassa Shear & B·O. Dodge	Copper chloride	10–20	Spherical	FTIR, TEM, SEM, EDX and XRD	100
Pestalotiopsis sp.	Copper chloride	10–20	Spherical	EDX, TEM, SEM, XRD and FTIR	100
Myrothecium gramineum Lib.	Copper chloride	10–20	Spherical	FTIR, TEM, SEM, EDX and XRD	100

#### 2.3.3 Copper oxide nanoparticles synthesised by bacteria

Recently, scientists have used microorganisms to manufacture nanoparticles like copper oxide.^86^ Bacteria have been used to make diverse materials with fascinating nanoscale dimensions and structures via intracellular and extracellular mechanisms. The capacity of microorganisms to generate nanoparticles is encouraging. They offer features like rapid generation, straightforward culture methods, secure experimental settings, excellent stability, the production of nanoparticles outside of cells, and ease of genetic manipulation. Microbes can adapt to high metal concentrations by converting harmful ions into less toxic substances, such as metallic sulphides and oxides. Bacteria have been recognised for transforming hazardous metal ions into less deadly oxides to survive in situations with significant levels of these metals (103–106).^101^, ^102^, ^103^ Bacteria synthesise numerous crucial thiol-containing chemicals as a defensive mechanism against oxidative stress. The bacterially induced production of nanoparticles acts as an encapsulating agent, preventing the oxidation of nanoparticles from metal oxide.^104^^,^^105^ We still don't know how the nanoscale transformation happens. Moderate experimental parameters, including temperature and pH, easy downstream processing and a brief formation period, are also necessary for nanoparticle generation.^106^ Table 3 presents a selection of the roles different bacteria play in forming nanoparticles made from copper oxide through bioremediation.

**Table 3:** Copper oxide nanoparticles synthesised by Bacteria.

Bacterial culture	Gram nature	Precursor	Size (nm)	Shape	Characterisation techniques	Reference
Actinomycetes	Gram-positive	Copper (II) sulphate	61	Spherical	UV–Vis, XRD, TEM, FTIR	52
Bacillus cereus	Gram-positive	Copper (II) sulphate	11–33	Spherical	TEM, SEM UV–Vis, FT-IR, and XRD	107
*Escherichia coli*	Gram-negative	Copper Sulphate	100–150	Quasi-spherical	UV–Vis TEM, AND SEM	101
Gluconacetobacter hansenii	Gram-negative	Copper Sulphate	25–35	–	TEM, XRF, SDD and EDX,	104
Lactobacillus casei subsp.	Gram-positive	Copper (II) sulphate	30–75	Spherical	UV–Vis TEM and SEM	105
Morganella sp.	Gram-negative	Copper (II) sulphate	15–20	Spherical	FTIR, TEM, Zeta Potential, and XRD	32
Mycobacterium psychrotolerans Trujillo and *Morganella morganii* RP42 Winslow	Gram-negative	Copper Sulphate	15–20	Quasi-spherical cubic	UV–Vis, XRD, TEM, FTIR	108
Pseudomonas fluorescens Migula	Gram-negative	Copper Sulphate	20–80	Spherical and hexagonal	TEM, SEM UV–Vis, FT-IR, and XRD	106
Pseudomonas stutzeri	Gram-negative	Copper Sulphate	8–15	spherical	UV–Vis TEM, AND SEM	109
Streptomyces sp. MHM38	Gram-positive	Copper sulphate	1.72–13.49	Spherical	TEM, XRF, SDD and EDX,	110
*Salmonella typhimurium*	Gram-negative	Copper sulphate	49	Spherical	UV–Vis, DLS, SEM	111
Shewanella loihica PV-4	Gram-negative	Copper chloride	6–20	cubic	TEM, EDX, XRD, XPS	112
Serratia sp.	Gram-negative	Copper sulphate	10–30	Polydisperse	TEM, SEM, EDS, UV–Vis,SPR, EDX	19

#### 2.3.4 Copper oxide nanoparticles synthesised by algae

The efficiency and accessibility of algae nanoparticle manufacturing have recently garnered attention. Algae extract contains bioactive compounds that have the potential to stabilise and reduce the size of nanoparticles.^113^ Comparing these substances to other biological resources like plants, bacteria, and fungi must be utilised appropriately. We can use algae, abundant in bioactive compounds, to synthesise nanoparticles of metals and metallic oxides.^114^ Various factors, such as algal biomass/extract concentration, growth duration, pH levels, salts, and reaction temperature, play a crucial role in determining the size and shape of metal oxide materials. A wide range of algae, such as Chlorella vulgaris, Bifurcaria bifurcate, Sargassum muticum, Spirulina platensis, Pithophora oogonia, Ecklonia cava, Chlamydomonas reinhardtii, Oscillatoria willie, and Sargassum wightii, can synthesise metal and metal oxide nanoparticles. Some of the nanoparticles they can produce include silver, gold, zinc oxide, and iron oxide.^115^^,^^116^ The manufacture of copper oxide nanoparticles by algae has received little attention. We still don't know much about the many organic components of the many different types of algae that use copper as a promoter for reduction and stabilisation. Therefore, it is crucial to research CuONPs using biomolecules in green synthesis to expand their biological applications. You can find them in Table 4.

**Table 4:** Copper oxide nanoparticles synthesised by Algae.

Algae culture	Precursor	Size (nm)	Shape	Characterisation techniques	Reference
Anabaena cylindrical Lemmermann	Copper Sulphate	3.6	Crystallite	XRD, XPS, EDX	117
Bifurcaria bifurcata R. Ross	Copper Sulphate	5–45	Crystallite	UV–Vis, TEM, FTIR, XRD	118
Botryococcus braunii Kützing	Copper Acetate	10–70	Cubical and Spherical	UV–Vis, FTIR, SEM, X-Ray Diffraction.	119
Cystoseira trinodis (Forsskål) C. Agard	Copper Sulphate	6–7.8	Crystallite	TEM, SEM, XRD, FTIR, EDX, Raman, UV–Vis	120
Macrocystis pyrifera (L.) C·Ag.	Copper Sulphate	2–50	Spherical	DLS, Zeta Potential, FTIR, TEM, EDS	121
Sargassum polycystum C. Agardh	Aqueous copper	-	Spherical	TEM, SEM, XRD, FTIR, EDX, UV–Vis	122

#### 2.3.5 Copper oxide nanoparticles synthesised by actinomycetes

Actinomycetes are a type of prokaryotic microorganism. Groundwater, freshwater, and marine settings commonly harbour these gramme-positive, spore-generating bacteria. Actinomycetes are an essential reservoir of secondary metabolites, including antimicrobial agents, nutrients, chemotherapy agents, herbicides, and immunosuppressive substances.^123^^,^^124^ Actinomycetes widely recognise the generation and various uses of nanoparticles. Actinomycetes synthesise nanoparticles through both intracellular and external paths inside cells; cations from carboxylate parts interact with precursors on the mycelia cell wall to form small nanoparticles. Nitrogen cycle enzymes synthesise nanoparticles in the extracellular pathway. We have yet to extensively research the secondary metabolites and synthesise several nanoparticles, including gold, zinc, manganese, and silver.^125^ We need better to document the production of copper oxide nanoparticles by actinomycetes.

Actinomycetes that reside within the leaves of Oxalis corniculata L. produce copper oxide nanoparticles that have a spherical shape, and the size of the nanoparticle is 80 nm. We treated the biomass filtrate from actinomycetes with CuSO4·5H2O at a temperature of 35 °C for 6 h. The colour of the biomass filtrate transitions from a pale blue hue to a greenish-brown shade, which suggests a decrease in the presence of metal ions and the creation of copper oxide nanoparticles. Further nanoparticle characterisations encompass using different analytical techniques and studies into their antimicrobial, antioxidant, and larvicidal characteristics. Green copper oxide nanoparticles showed a lot of biological activities, such as killing microbes, protecting cells from damage, and killing chicks and larvae of *Musca domestica* and *Culex pipiens*.^126^ The researchers, Nabila and Kannabiran (2018), used actinomycetes to make consistently round copper oxide nanoparticles with an average diameter of 61.7 nm. Copper oxide nanoparticles from actinomycetes are produced with a water-based solution of copper sulphate. The resulting mixture was then subjected to a temperature of 100 °C for 15 min. The filtrate had a colour change from blue to reddish brown. The characterisations were validated using ultraviolet (UV) spectroscopy, XRD, FTIR, TEM, SEM, DLS, EDX, and zeta potential analysis. Secondary bioactive metabolites are essential for stabilising the nanoparticles and biogenic production. The functional group on the copper oxide nanoparticles, a secondary metabolic compound of actinomycetes, is responsible for encapsulation, reduction, stabilisation, and synthesis, as found in the FTIR study. The bioinspired nanoparticles were tested and found to have strong antibacterial properties against various human pathogenic pathogens.^52^

### 2.4 Recent advances in the synthesis of copper oxide nanoparticles

Copper oxide nanoparticles have garnered significant attention due to their unique properties and diverse applications in fields such as catalysis, electronics, energy storage, and biomedicine.^127^ Traditional synthesis methods, including sol-gel, hydrothermal, and co-precipitation, have been widely employed to produce CuO nanoparticles.^128^ However, these methods often suffer from limitations such as long reaction times, high costs, and environmental concerns.^129^ In recent years, novel synthesis techniques have emerged, offering enhanced efficiency, cost-effectiveness, scalability, and reduced environmental impact. This manuscript provides the latest developments in CuO nanoparticle synthesis, focusing on four innovative methods: green microwave-assisted synthesis, microfluidic synthesis, ultrasound-assisted chemical reduction, and photochemical synthesis.

#### 2.4.1 Green microwave-assisted synthesis

Green microwave-assisted synthesis represents a sustainable approach to CuO nanoparticle synthesis by utilising eco-friendly reducing agents and microwave heating.^130^ Natural extracts, such as those derived from plants like Moringa oleifera, serve as reducing agents, facilitating the rapid formation of CuO nanoparticles under microwave irradiation. This method offers several advantages, including reduced synthesis time, improved energy efficiency, and enhanced biocompatibility of the nanoparticles.^131^

CuO NPs can be synthesised in one pot utilising microwave irradiation and apple peel extract as a nontoxic reducing and capping agent. No hazardous reagents or precursors are used in this simple process. Synthesised NPs undergo analysis using FE-SEM, HR-TEM, XRD, XPS, and Raman. Additionally, green CuO NPs significantly reduce MB and CV dyes.^132^

#### 2.4.2 Microfluidic synthesis

Microfluidic synthesis provides precise control over reaction parameters by employing microreactors to manipulate the reaction environment.^133^ Copper salt solutions and reducing agents are pumped through microfluidic channels, where they mix and react to form CuO nanoparticles. This technique enables fine-tuning of particle size and morphology, high reproducibility, and scalability through parallelization of microreactors.^134^

#### 2.4.3 Ultrasound-assisted chemical reduction

Ultrasound-assisted chemical reduction harnesses ultrasonic waves to accelerate the synthesis of CuO nanoparticles.^135^ By applying ultrasonic waves to a solution containing copper salts and reducing agents, the reduction reaction is promoted, leading to the formation of CuO nanoparticles with enhanced properties. This method offers advantages such as increased reaction rates, improved particle dispersion, and energy efficiency.

#### 2.4.4 Photochemical synthesis

Photochemical synthesis utilizes light energy to drive the reduction of copper ions to CuO nanoparticles.^136^ A photocatalyst, such as titanium dioxide (TiO2), is employed to absorb light and generate reactive species that facilitate the reduction reaction. This method enables the synthesis of nanoparticles under mild conditions, preserving the functional properties of sensitive biomolecules, and offers environmental benefits by avoiding the use of harmful chemicals.

#### 2.4.5 Comparative analysis with traditional techniques

The new synthesis methods offer significant improvements over traditional techniques in terms of efficiency, cost-effectiveness, scalability, and environmental impact. Green microwave-assisted synthesis and ultrasound-assisted chemical reduction reduce reaction times, while microfluidic synthesis allows for continuous production and scalability. These techniques utilize natural extracts, inexpensive materials, and energy-efficient processes, resulting in reduced costs and environmental footprint compared to traditional methods. Photochemical synthesis offers precise control over reaction conditions and avoids the use of hazardous chemicals, further enhancing its environmental sustainability.

## 3 Characterisation of copper oxide nanoparticles

Researchers should conduct a series of characterisations to ascertain the necessity for nanoparticle synthesis. After synthesising NPs, the first step in characterisation is to choose their crystal shape and chemical composition.^137^ The chemical construction of the CuONPs was examined using multiple techniques, such as ultraviolet–visible spectroscopy, X-ray scattering, surface plasmon resonance, FT-IR, and energy-dispersive X-ray spectroscopy. The dimensions, like the shape and size of the CuONPs, were analysed using transmission and scanning electron microscopy, dynamic light scattering, zeta particle analysers, and field-emission scan electron microscopy (Table 5).

**Table 5:** Different characterisation techniques for copper oxide nanoparticles.

Techniques	Properties	Parameters
UV visible spectroscopy	NP formation	Confirmation of the synthesis
Scanning tunneling microscope	Size and morphology analysis	Topology and chemical analysis
Scanning electron microscope	Size and morphology analysis	Topology, size, morphology, crystallographic structure
Transmission electron microscope	Size and morphology analysis	Topology, size, morphology and crystallographic structure
Atomic force microscope	Size and morphology analysis	Size, morphology, surface roughness and texture
Dynamic light scattering	Size and morphology analysis	Amorphous contents and polymorphism
Differential scanning calorimetry (DSC)	Analysis of heat	Polymorphism and Amorphous
Fourier transmission infrared spectroscopy	Functional group analysis	Identification of functional groups
Zeta potential	Surface analysis	Colloidal stability and surface charge
Energy dispersive X-ray	Elemental analysis	Chemical composition and purity
Particle size analysis (PSA)	size analysis	To measure the distribution of size
X-ray absorption spectrometry	Elemental analysis	Electronic structure and elemental composition
X-ray fluorescence spectroscopy	Elemental analysis	Chemical composition and thickness of coating

### 3.1 X-ray diffraction

Characterizing CuO nanoparticles (NPs) is essential for tailoring their properties to specific applications. One of the most widely used methods is X-ray Diffraction (XRD), which determines the crystalline structure and phase purity. XRD works by diffracting X-rays through the crystal lattice of the material, producing a pattern characteristic of its structure. This method is highly accurate and non-destructive, providing detailed information on crystallite size using Scherrer's equation.^138^ However, it requires well-prepared, crystalline samples and is less effective for amorphous materials. For instance, in catalysis, the efficiency of CuO nanoparticles can be influenced by their crystallinity, making XRD crucial for ensuring the correct phase of CuO (monoclinic) is present. In sensor applications, the correct crystalline phase can enhance sensitivity and response time.

### 3.2 Transmission electron microscopy

Transmission Electron Microscopy (TEM) offers high-resolution images to analyze morphology, size, and structure. TEM transmits electrons through an ultra-thin sample, interacting with the material to form detailed images down to the atomic level. This method provides precise information on size distribution, shape, and defects, which is crucial in biomedical applications where the uniformity of CuO NPs impacts biocompatibility and effectiveness.^139^ However, TEM requires extensive sample preparation and operates in high vacuum conditions. For drug delivery, controlling particle size and shape, visible through TEM, can influence the release profile and targeting ability of CuO NPs.^140^

### 3.3 Scanning electron microscopy

Scanning Electron Microscopy (SEM) examines surface morphology and composition by scanning a focused electron beam across the sample surface, causing the emission of secondary electrons to form an image. SEM offers high depth of field and surface detail and can be complemented with Energy Dispersive X-ray Spectroscopy (EDX) for compositional information.^141^ It is particularly useful for revealing the porous structure of CuO NPs in gas sensors, which is vital for their sensitivity and adsorption properties. However, SEM is limited to surface imaging and requires careful sample preparation. In catalysis, SEM helps analyze surface roughness and active sites, which are crucial for reaction mechanisms.

### 3.4 Fourier Transform Infrared Spectroscopy

Fourier Transform Infrared Spectroscopy (FTIR) identifies chemical bonds and functional groups by passing infrared light through the sample, where bonds absorb at characteristic frequencies. FTIR is non-destructive, fast, and provides information on chemical composition and surface modifications.^142^ However, it is limited to identifying functional groups rather than the entire molecular structure. For example, FTIR can confirm the presence of surface modifications on CuO NPs intended for drug delivery systems, ensuring biocompatibility. In biomedical applications, verifying the presence of biocompatible coatings with FTIR is essential.^143^

### 3.5 Dynamic light scattering

Dynamic Light Scattering (DLS) measures particle size distribution in colloidal suspension by analyzing fluctuations in light scattering due to Brownian motion of particles. DLS is quick and relatively easy to use, making it effective for measuring nanoparticles in suspension. However, it can be affected by agglomeration and polydispersity. DLS ensures uniform size distribution of CuO NPs in water treatment applications, where size consistency impacts filtration efficiency. In medical diagnostics, precise particle size measured by DLS ensures reproducibility and accuracy in assays.^139^

### 3.6 X-ray Photoelectron Spectroscopy

X-ray Photoelectron Spectroscopy (XPS) determines surface elemental composition and oxidation states by exciting core electrons with X-rays, measuring the emitted electrons' kinetic energy. XPS is sensitive to surface chemistry and provides quantitative elemental analysis. However, it is expensive, requires high vacuum, and is surface-sensitive (typically only the top 10 nm).^144^ XPS can confirm the oxidation state of Cu in CuO NPs for battery applications, ensuring correct electrochemical properties. For catalytic applications, surface composition analysis by XPS is critical to understanding active sites.

### 3.7 Atomic force microscopy

Atomic Force Microscopy (AFM) provides 3D surface morphology at the nanometer scale by scanning a sharp tip across the sample surface, with deflections forming a topographical map. AFM offers high-resolution 3D imaging and can operate in various environments (air, liquid). However, it is limited to surface analysis and has a relatively slow scanning process. AFM can reveal the surface texture of CuO NPs in photovoltaic cells, impacting light absorption efficiency.^145^

### 3.8 Raman Spectroscopy

Raman Spectroscopy identifies molecular vibrations, providing structural and compositional information through the inelastic scattering of monochromatic light (Raman effect). It is non-destructive and suitable for a wide range of materials, though fluorescence from some materials can interfere with the Raman signal. Raman spectroscopy can study the interaction of CuO NPs with biological molecules in biomedical applications.^146^

## 4 Biomedical application of copper oxide nanoparticles

**Fig. 3:**
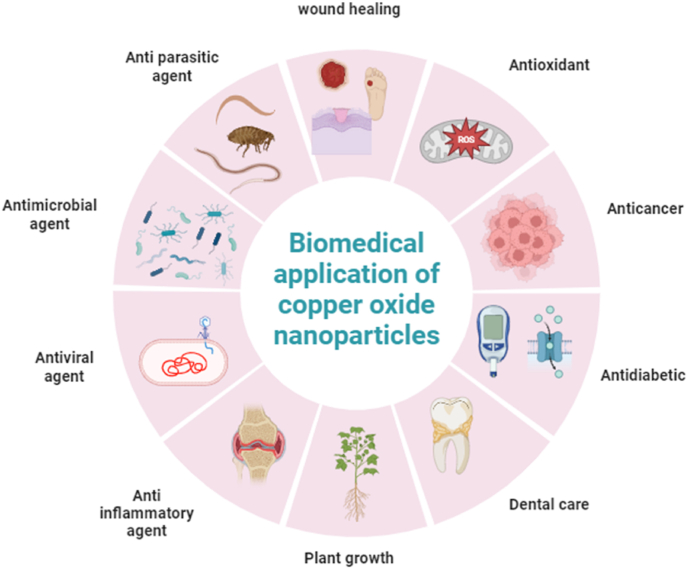
Biomedical application of copper oxide nanoparticles.

Copper nanoparticles have a wide range of biological uses. They demonstrate a significant effectiveness against a broad spectrum of disease-causing microorganisms. Elevated concentrations of CuONPs induce the production of reactive oxygen species within bacterial cells, resulting in the breakdown of cell membranes. CuONPs have demonstrated both anticancer and antifungal effects. Its antimicrobial characteristics make it appropriate for food preservation and use in agricultural applications. To develop a defensive mechanism against specific pathogenic microorganisms like algae, fungi, and bacteria.^147^ Copper-based nanofertilizers and nano-insecticides enhance the growth and nutrient uptake of crop plants. Copper-based biological remediation is crucial for treating wastewater and eliminating heavy metals in soil. Copper possesses high electrical conductivity, rendering it highly valuable in contemporary electronics as a superconductor. Fig. 3 summarises the various applications of CuONPs and specifically details their primary application.^148^

### 4.1 Copper oxide nanoparticle antimicrobial activity

One of the leading causes of human suffering on a global scale is infectious diseases caused by microorganisms. Scientists are attempting to come up with novel approaches to combat microbial diseases. The emergence of resistance towards conventional treatments, especially antibiotic resistance, which restricts the effectiveness of standard medicines in fighting microbial diseases, is a significant reason why research is ongoing. Researchers investigated nanomaterials as a potential new antibacterial agent due to the widespread efforts to control and prevent diseases caused by microbes.^149^ In recent decades, metallic oxide-based nanoparticles have effectively combated numerous bacterial and viral diseases. Because they are less harmful, more environmentally friendly, and may be able to cure diseases, medicines based on nanoparticles have attracted a lot of attention. Multiple studies have shown that CuONPs exhibit potent antibacterial properties against harmful bacteria. CuONPs poison many plants and humans at high concentrations.^150^ CuONPs exhibit potent antibacterial properties due to their diminutive dimensions, expansive surface area, compatibility with living organisms, and elevated biochemical and physical reactivity levels. In extensive laboratory testing, CuONPs have demonstrated promising antibacterial activity against various human pathogenic bacterial strains.^151^^,^^152^ The potential of CuO NPs as disinfectants, agents in food processing, and components of medical devices is also a topic of active investigation.^153^ Research into the specific process by which antimicrobials work continues.

### 4.2 Copper oxide nanoparticles antifungal activity

Fungal diseases have become a significant issue in current medicine, particularly for patients with compromised immune systems, such as those with immunodeficiency or cancer patients receiving chemical treatments for their disease.^154^^,^^155^ Medical researchers have looked into the possibility that CuONPs can fight fungal infections due to their antifungal properties.^156^^,^^157^ It has been documented that the Penicillium chrysogenum fungal strain can promote the formation of copper oxide nanoparticles. Penicillium citrinum, Aspergillus niger, Fusarium oxysporum, and Alternaria solani were among the harmful bacterial and fungal strains that the scientists tested these environmentally synthesised nanoparticles against. In addition, it was found that the environmentally friendly production of copper oxide nanoparticles had positive effects on Alternaria solani, Fusarium oxysporum, *A. niger*, and Penicillium citrinum. The inhibition zones for these organisms were measured at 37.0 ± 0.76376, 26.5 ± 0.76376, 28.0 ± 0.86603, and 20.7 ± 0.43589, respectively.^138^ Also, utilising Syzygium alternifolium extract, Berra et al. (2018) showed that CuONPs may be synthesised in an environmentally friendly way.^135^ Table 6 shows that Cu/CuO NPs have antifungal characteristics, which means they could be used to treat fungal infections. A. Eriophyllum was used to synthesise CuONPs, according to a study. Compared to bacteria, boiss extract of leaves is more resistant to fungi like Candida guilliermondii and Candida krusei.^117^ The fungus cell wall contains multiple lipid layers, making it difficult for NPs to enter the organism.^87^ Recent research has shown that A. flavus can be treated with CuONPs by green synthesis, which has antifungal effects by damaging the outer layer of cells and accumulating reactive oxygen species (ROS).^65^ Moreover, their antioxidant activity may be attributed to the antifungal action of the CuO NPs derived from A. sativum extract.^117^^,^^158^

**Table 6:** Copper oxide nanoparticles Antifungal activity.

Fungal species	Precursor	application	Reference
Cissus quadrangularis L.	Cu (CH_3_COO)_2_	Suppress the proliferation of harmful fungi Aspergillus niger Tiegh and Aspergillus flavus Link.	91
Syzygium aromaticum (L.) Merr. & L.M.Perry	CuSO_4_	Demonstrated fungicidal properties against harmful fungi. A. flavus Link and *A. niger* Tiegh.	156
Brassica juncea (L.) Czern.	CuSO_4_	inhibit the development of Phoma destructiva, Curvularia lunata, and *Alternaria alternata*.	91
Oxalis corniculata L.	CuSO_4_	Suppress the proliferation of Pythium ultimum Trow, *A. alternata*, and *A. niger* Tiegh.	126
Syzygium alternifolium (Wight) Walp.	Cu(NO_3_)_2_	Efficient in combating Trichoderma harzianum Rifai	154
Citrus Medica L.	CuSO_4_	Potent against disease-causing fungi are F. oxysporum Schltdl., Fusarium graminearum Schwabe, and F. culmorum Sacc.	155
Penicillium chrysogenum Thom	CuSO_4_	Antifungal action specifically targeting A. solani L.R. Jones, Aspergillus niger Tieghem, and F. oxysporum Schltdl.	157

### 4.3 Copper oxide nanoparticles antibacterial activity

Recently, nanotechnology therapies have been used to diagnose and treat diseases and develop new medications. The efficacy of nanoparticles in inhibiting the growth of various harmful bacterial strains has been assessed, with significant results.^159^^,^^160^ Based on literature investigations, it has been found that CuONPs exhibit significant toxicity towards several human illnesses.^150^ The use of fabrication to manufacture CuONPs has attracted considerable interest due to their potential as an antibacterial agent due to their desired morphologies, like shape and size, and compatibility with biological systems, which makes them highly efficient in controlling many harmful human bacteria.^57^^,^^161^^,^^162^ The synthesised CuONPs, exhibiting a green hue, exhibit strong antibacterial properties against gram-positive and dangerous strains. The increased antibacterial efficacy of CuONPs has been linked to the presence of a secondary metabolite compound in the extract used during the encapsulation process.^163^ The antibacterial evaluation of CuONPs obtained using the agar well diffusion method demonstrated their harmful effects in inhibiting the growth of tested pathogens, including gram-positive organisms (*Staphylococcus aureus* and Streptococcus mutans) as well as gram-negative bacteria (*Pseudomonas aeruginosa*, *Klebsiella pneumoniae*, and *Escherichia coli*). CuONPs generated from plants effectively suppressed the development of Gram-negative bacteria commonly associated with urinary tract infections, such as Enterococcus sp., *Escherichia coli*, Klebsiella sp., and Proteus sp.^164^ This demonstrates the nanoparticles' ability to treat these illnesses effectively. Plant-based nanoparticles (NPs) can inhibit the replication of K. pneumonia, a pathogenic bacterium that can cause injury if it disseminates to other body parts. Copper (Cu) and copper oxide (CuO) nanoparticles have been shown to inhibit the growth of Gramme-positive bacteria, notably B. subtilis, S. pyrogens, and *S. aureus*, which is a pathogenic microbe that causes respiratory and skin illnesses, whereas B. subtilis can cause diarrhoea. On the other hand, S. pyrogens specifically targets humans and leads to a range of diseases, including rheumatic fever and scarlet fever.^165^ Table 7 summarises the reports on the antibacterial activity of CuONPs.

**Table 7:** Antibacterial effects of plant-biosynthesised CuO NPs.

Bacterial Species	Size (nm)	Shapes	Concentration	Diameter of Inhibition Zone (mm)/Inhibition (%)	Reference
Campylobacter coli	48–76	Globular	25 μg/mL	20	169
*Escherichia coli*	5–20	Spherical	100 μg/mL	18	147
	10–30	Spherical	4 mg/mL	12.1	170
	48–76	Globular	25 μg/mL	18	169
Moraxwlla catarrhalis	48–76	Globular	25 μg/mL	24	169
*Pseudomonas aeruginosa*	10–30	Spherical	4 mg/mL	13.8	170
Salmonella Typhimurium	10–30	Spherical	8 mg/mL	16.8	170
Vibrio harveyi	18.9–45.2	Spherical	5 μg/mL	98 %	72
Klebsiella	16.8	Spherical	3 mg	12	58
*Proteus mirabilis*	10–30	Spherical	8 mg/mL	13.2	170
Bacillus subtilis	10–50	Spherical	50 μg/mL	15	171
Bacillus cereus	5–22	Spherical	MIC:21 μg/mL	–	166
Listeria monocytogenes	48–76	Globular	25 μg/mL	9	169
Micrococcus luteus	67–99	Spherical	10 μL of 170 mL of 1 mM	23.33	73
			CuSO4_5H2O aqueous		
			solution + 30 mL of 1 % green tea extract		
Staphylococcus saprophytic	10–30	Spherical	2 mg/mL	12.4	170
Streptococcus pyrogens	20–40	Spherical and oval-shaped	50 μg/mL	3.05	165
Streptococcus mutans	20	Spherical	1000 μg/mL	13	80

#### 4.3.1 Hypothesised mechanism for antibacterial action

**Fig. 4:**
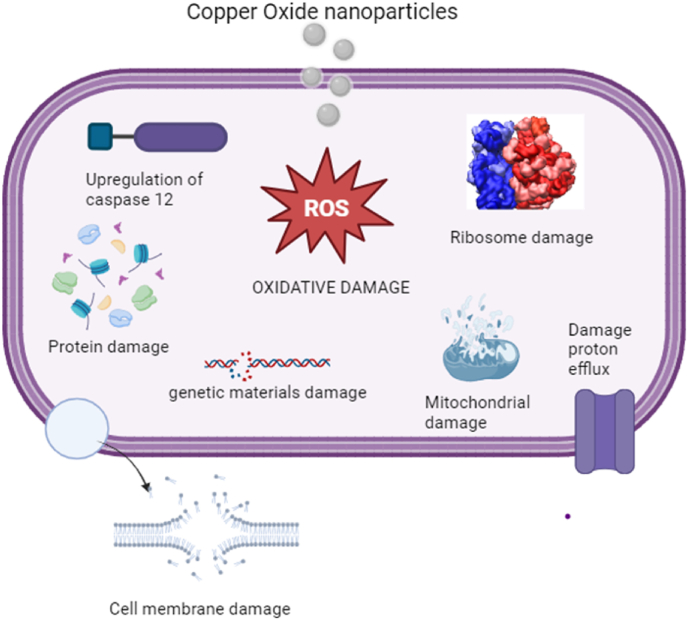
Probable antibacterial mechanism of copper oxide nanoparticles.

In general, the CuONPs can enter the bacterial cells by engaging with the cell membrane, allowing the Cu2+ ions to pass through.^56^ Field emission scanning electron microscopy (FESEM) was employed to observe the altered morphology of bacterial cell membranes upon exposure to CuONPs produced using an aqueous fruit-dried extract of *T. terrestris*. The CuONPs stimulated the production of the reactive oxygen species ROS, which was responsible for the antibacterial effect (Fig. 4).^166^ Several plant-based CuONPs have been found to possess both antibacterial and antioxidant activities.^167^ The study found that C. Virginia-mediated copper oxide nanoparticles had antioxidant activity, which hinders the growth of bacteria, causing urinary tract infections.^164^ The antibacterial effects of copper oxide and copper nanoparticles obtained from preparations of the sativum Allium and Allium eriophyllum Boiss leaf are likely due to their antioxidant properties.^167^^,^^168^

### 4.4 Copper oxide nanoparticles antiviral activity

There has been limited research on the antiviral properties of copper oxide nanoparticles synthesised using green methods. These studies have demonstrated that the nanoparticles made of copper oxide also possess significant antiviral activity.^154^ In their research, P. Yugandhar et al. described the production of CuONPs using Syzygium alternifolium fruit juice as a green mediator. The researchers confirmed the synthesis of the nanoparticles by various characterisation techniques, including DLS (dynamic light scattering), XRD (x-ray diffraction), AFM (atomic force microscopy), SEM (scanning electron microscopy), and TEM (transmission electron microscopy). The synthesised nanoparticles exhibited a spherical morphology with an average diameter of 17.5 nm. The scientists used the haemagglutination (HA) test to determine how well copper oxide nanoparticles could fight the Newcastle Disease Virus (NDV). The study revealed that the artificially created bioinspired copper oxide nanoparticles demonstrated potent antiviral properties. This discovery contains exciting possibilities for therapeutic uses and the development of novel drugs.^154^ Cui et al. (2021) conducted a study incorporating CuONPs into electrospun nanofibers with PVP to enhance virus elimination, the virus known as H1N1 was used as subject material to demonstrate its antiviral effects. After being exposed to CuONPs for 4 h, nearly 70 % of the infections remained inactive.^172^ These findings indicate that CuONPs possess substantial antiviral efficacy. Researchers have found that these nanoparticles possess exceptional antiviral effects, as supported by multiple investigations,^173^ amidst the global pandemic caused by SARS-CoV-2, resulting in numerous fatalities. Researchers recorded the accumulation of several widely recognised antibacterial substances on flat, solid surfaces and permeable filter materials. The researchers evaluated the efficacy of their antiviral properties against the ability of SARS-CoV-2 to survive, and they linked them to virus plaque assays.^174^

### 4.5 Copper oxide nanoparticles antiparasitic activity

Various metal oxide nanoparticles have been studied to assess their efficacy against different parasites, yielding positive results.^175^^,^^176^ The antimalarial and antimycobacterial capabilities of CuONPs produced from Acanthospermum hispidum were evaluated in vitro against the *Mycobacterium tuberculosis* and P. falciparum strains (H37RV). The CuONPs demonstrated significant antimalarial efficacy against Plasmodium falciparum, with an acceptable MIC of 1.08 μg/mL. This activity was superior to the traditional antimalarial drugs chloroquine and quinine, which had MICs of 0.020 μg/mL and 0.268 μg/mL, respectively. Furthermore, the CuO nanoparticles completely stopped the growth of Mycobacterium TB H37RV at a MIC of 100 μg/mL.^177^ The study effectively generated CuO, NiO, and Cu/Ni hybrid nanoparticles utilising an extract derived from Curcuma longa roots in an environmentally friendly manner. Cu/Ni hybrid nanoparticles were the most effective at killing promastigotes and amastigotes when diluted to 400 μg/mL, with numbers of 60.5 ± 0.53 for promastigotes and 68.4 ± 0.59 for amastigotes. In comparison, the NiO NPs showed lower activity with values of 53.2 ± 0.48 and 61.2 ± 0.44, while the CuO-NPs had a value of 56.2 ± 0.45.^178^

### 4.6 Wound healing and anti-inflammatory activity of copper oxide nanoparticles

Recent studies have shown that CuONPs can protect wounds from infections and aid healing. Many harmful germs get into the body, mainly through cuts, scrapes, and other visible wounds and scars. Removing germs from the wound site is critical for infection prevention. As previously mentioned, CuONPs exhibit promising antibacterial and antifungal properties, making them suitable for wound healing applications.^179^ Copper (Cu) stimulates angiogenesis, the process of vascular endothelial growth, close to the site of the wound. In addition, CuONPs promote the synthesis of vascular endothelial growth factor (VEGF), a crucial element in wound healing and a catalyst for the transportation of nutrients.

Furthermore, it stimulates the growth of cells, facilitates their movement, and hinders the activity of the cyclooxygenase-2 enzyme at the location of the wound.^107^^,^^180^ The application of CuONP therapy mediated by Abies spectabilis successfully decreased the levels of inflammatory mediators and leukocytes in an animal model of inflammatory pain induced by various stressors.^181^ Creating CuONP medication from plants like A. eriophyllum and F. ananassa has revealed benefits for wound healing. This is demonstrated by decreased recruitment of neutrophils and lymphocytes and the closure of cutaneous wounds in rats.^167^ Copper nanoparticles were made with extracts from Falcaria vulgaris leaves that seemed like they might help wounds heal.^182^ An incision measuring 500 mm was performed on an anaesthetised animal as part of a research investigation. A gel was created to create three configurations: one with biologically synthesised CuNP, one without, and a control. A few days later, it was shown that biologically synthesised CuONPs could reduce the damage by 92 %. It has been documented that A. sativum CuO NPs can effectively reduce inflammation caused by egg albumin.^168^ These studies show that CuONPs can be synthesised in a green way and have the potential to reduce inflammation, ease pain, and speed up the healing process after wounds. However, new research using macrophages has shown that NLRP3 inflammasome activation is possible using marketable CuONPs and their ions. The inflammasome is a biological sensor that controls cytokine production that stimulates inflammation.^183^ This recent discovery indicates that the phytochemicals included in plant-mediated CuONPs can inhibit the inflammatory response.

### 4.7 Antioxidant activity of copper oxide nanoparticles

The green-synthesised CuONPs have demonstrated significant antioxidant activity. Scientists have employed many innovative methods to verify and measure the antioxidant properties of CuONPs synthesised using green methods.^58^ Researchers have utilised various techniques, such as the DPPH radical scavenging test, ferrous reduced antioxidant power, or FRAP assay, total antioxidant assays, ferric thiocyanate (FTC) assay, and the total phenolic content, or TPC, assay, to assess the antioxidant efficacy of CuONPs synthesised by green methods. A study by Ruiz et al. showed that CuONPs activated catalase (CAT) and superoxide dismutase (SOD) in different parts of *Mytilus galloprovincialis* mussels.^184^ In a different study, researchers found that adult female rats have antioxidant enzyme activity, specifically *Rattus norvegicus* var. Albinos when exposed to CuO NPs. CAT, SOD, glutathione S-transferase (GST), glutathione peroxidase (GPx), and glutathione reductase (GR) levels were quantified in the rat liver.

Studies conducted using transmission electron microscopy (TEM) showed that after CuO NPs were applied, there were significant changes in the liver's antioxidant enzymes.^185^ Vinothkanna et al. synthesised CuONPs using the bark of Rubia cordifolia and evaluated their antioxidant capacity.

A study was done to produce nanoparticles of copper oxide from Andean blackberry fruits (ABF) and leaves (ABL). The generated nanoparticles were then analysed to determine their antioxidant capabilities.^186^ The findings demonstrated that CuONPs facilitated by ABF exhibited a more excellent antioxidant activity (89.02 %) than CuONPs facilitated by ABL, which displayed an estimated 75 % in the DPPH assay at one mM.^187^ Asghar et al. produced CuONPs from petals from Rosa foetida (Rf), Rosa kardina (Rk), and Rosa delicia (Rd) roses. The roses of CuONPs have sizes of 17.7, 26.3, and 14.9 nm. The CuONPs from RK had a maximal antioxidant potential of 98.07 μg AAE/mg and a reduction capacity of 73.50 μg AAE/mg. RK CuONPs had the most excellent DPPH scavenging activity, 60.9 %.^188^

### 4.8 Anticancer and cytotoxicity of copper oxide nanoparticles

Globally, cancer is a prominent contributor to mortality. Various therapeutic modalities, including chemotherapy, radiation therapy, and surgery, have been employed for the treatment of cancer. However, these approaches are limited by their high cost and potential adverse effects. Hence, there is a requirement for a treatment that is both non-toxic and cost-effective while also having minimal adverse effects.^154^^,^^189^ Nanoparticles are significantly smaller than more giant biological molecules like enzymes and receptors. They also exhibit distinctive form and stability and provide exclusive contact with biomolecules. These characteristics can potentially aid in the treatment and diagnosis of cancer.^105^, ^190^ Various nanomaterials have been shown to prevent cancer cell proliferation.^191^ CuONPs and other types of nanoparticles have demonstrated promising anticancer properties against several types of cancer cells, as indicated in Table 8. To assess the anticancer properties of CuONPs, tumour cells were cultured at a temperature of 37 °C and exposed to a constant 5 % carbon dioxide concentration within the specified growth medium. The cells were organised in a plate containing 96 wells to facilitate exposing them to the nanoparticles.^192^^,^^193^ There are multiple colourimetric assays used to investigate the toxicity of cancer cells, such as 3-(4,5-dimethylthiazol-2-yl)-5-(3-carboxymethoxyphenyl)-2-(4-sulfophenyl)-2H-tetrazolium-MTS, 3-(4,5-dimethylthiazol-2-yl)-2,5-diphenyl tetrazolium bromide-MTT, and 4-[3-(4-Iodophenyl)-2-(4-nitrophenyl)-2H-5-tetrazolio]-1,3-benzenesulfonate-WST. These assays measure the metabolic processes of living cells by quantifying the level of activity of the reductase enzyme in the mitochondria.^194^ The anticancer activity of CuONP is accomplished through multiple mechanisms, such as the initiation of oxidative stress, the buildup of ROS, abnormalities in chromosomes, subdivision of genes, the synthesis of caspases, and the activation of intrinsic and extrinsic apoptotic pathways.^195^ Applying aqueous caviar criolla herbal extract resulted in the production of CuONPs. These nanoparticles exhibited a notable decrease in the cervical cancer cells (HeLa) using SRB cytotoxicity (see Table 9).

**Table 8:** Anticancer effects of the plant-derived Cu and CuO NPs biosynthesised.

Types of Cells/Cell Line	Size (nm)	Shapes	Toxicity (IC50) (μg/mL)	Biological Function (Targeting)	Reference
Breast cancer MCF-7	20	Spherical	24.5	ROS generation and antiangiogenic	203
MDA-MB-231	10–50	Spherical	30	ROS generation	171
Colon cancer HT-29	10–40	Spherical	33.0	Growth inhibition	204
Gastric cancer AGS	5–22	Spherical	25–50	Apoptosis	166
Ovarian cancer SKOV-3	20–50	Spherical	2.27	Antioxidant, loss of membrane potential, and DNA fragmentation	81
Cervical cancer HeLa	12	Spherical	26.73–20.32	Antioxidant and apoptosis	71
Leukaemia MOLT-4	10–40	Spherical	>80	Growth inhibition	204
Epithelioma Hep-2	12	Spherical	21.66–29.58	Antioxidant and apoptosis	71
Lung cancer A549	20	Spherical	81.57	Growth inhibition	80
Liver cancer HepG2	23–57	Spherical, hexagonal, cubical	>500	Antioxidant	74

**Table 9:** Performance Comparison: CuO Nanoparticles vs. Other Metal Oxides.

Activity	CuO Nanoparticles	ZnO Nanoparticles	TiO2 Nanoparticles
Antimicrobial Activity	CuO nanoparticles are highly effective against a broad spectrum of bacteria and fungi due to their strong oxidative stress-inducing capabilities and disruption of microbial cell membranes.	ZnO nanoparticles also possess significant antimicrobial activity, primarily through ROS generation and the release of Zn2+ ions, which can damage microbial cell membranes and interfere with metabolic processes. ZnO nanoparticles are particularly effective against Gram-positive bacteria.	TiO2 nanoparticles exhibit antimicrobial properties mainly when activated by UV light, which generates ROS. This photocatalytic activity can kill a wide range of microorganisms, but their efficacy under normal light conditions is limited.
Cytotoxicity and Biocompatibility	While effective in biomedical applications, CuO nanoparticles can exhibit higher cytotoxicity to mammalian cells due to ROS generation and potential DNA damage. Surface modification can mitigate these effects, enhancing biocompatibility.	ZnO nanoparticles generally show moderate cytotoxicity, which is dose-dependent and related to ROS generation and Zn2+ ion release.	TiO2 nanoparticles are typically considered to be highly biocompatible with low inherent cytotoxicity. However, their effectiveness in biomedical applications often requires UV activation, which can limit their practical use.
Photocatalytic and Antioxidant Properties	CuO nanoparticles have limited photocatalytic activity compared to ZnO and TiO2.	ZnO nanoparticles exhibit strong photocatalytic properties and are used in applications requiring ROS generation under UV light, such as in photodynamic therapy.	TiO2 nanoparticles are renowned for their exceptional photocatalytic activity, particularly under UV light. They are used in environmental applications and in photodynamic therapy for cancer treatment.
Drug Delivery and Tissue Engineering	CuO nanoparticles are promising for drug delivery due to their ability to generate ROS and induce cell death selectively in cancer cells. They can also enhance the mechanical properties and bioactivity of scaffolds in tissue engineering	ZnO nanoparticles are used in drug delivery systems for their biocompatibility and ability to enhance drug solubility and stability. In tissue engineering, they support cell proliferation and differentiation.	TiO2 nanoparticles are used in drug delivery, especially in UV-activated systems for controlled release. In tissue engineering, they provide structural support and enhance the mechanical strength of scaffolds.

Furthermore, minor alterations were found in the structure of mitochondria.^70^ In a separate investigation, CuONPs synthesised using an extract derived from the leaves of Pterolobium hexapetalum exhibited enhanced toxicity against the human breast carcinoma cell line (MDA-MB-231).^171^ Furthermore, it was shown that a chitosan/copper oxide nanocomposite, synthesised biologically with the aid of a bioflavonoid called rutin, demonstrated significant antiproliferative efficacy when evaluated against the human lung tumour cell line- A549.^196^ The anticancer action of CuONP has been observed in various types of cancer cells, including Mcf-7 breast cancer cells, CaCO-2 human colon cancer cells, HepG-2 hepatic cancer cells, and HeLa cells.^197^

An investigation disclosed that applying copper nanoparticles to the cells of HeLa caused the disintegration of mitochondria due to oxidative stress. The control cells demonstrate a characteristic mitochondria framework, but the treated tumour cells reveal a condensed and clustered organisation of mitochondria. This structural change ultimately triggers apoptosis, or programmed cell death, in the tumour cells.^70^ The investigation on the HepG-2 liver cancer cell line showed that apoptosis was enhanced by increasing the expression of the tumour suppression gene and reducing the levels of the antiapoptotic protein bcl-2.^198^

To further clarify this finding, it is essential to recognise that the toxicity of naturally occurring nanoparticles can be influenced by various factors, including their size, arrangement, morphology, and surface composition. Changes in multiple parameters can result in distinct cytotoxic reactions.

Moreover, biomolecules play a crucial role in the biosynthetic process of converting metal ions into their nanoscale counterparts. Biomolecules attach to the exterior layers of the nanoparticle that have been generated by biological synthesis and function as a stabiliser by inhibiting the aggregation of the nanoparticles. The presence of biomolecules attached to nanoparticles can modify the external chemistry of specific nanoparticles and impede their capacity to react to their biological surroundings. The biological sources from which nanoparticles are derived can impact their cytotoxicity.^199^, ^200^, ^201^, ^202^

### 4.9 Antidiabetic activity of copper oxide nanoparticles

Metal oxide nanoparticles have demonstrated significant promise in combating diabetes.^205^ Antidiabetic properties of CuONPs synthesised using an extract from Bacopa monnieri leaves. 34.4 nm nanoparticles of copper dioxide effectively reduced the blood glucose levels in animals with streptozotocin-induced diabetes.^206^ The results showed a reduction of 32.19 % and 33.66 % in blood glucose in rats treated with CuONPs and CuONPs/insulin combination, respectively. The antidiabetic effect of Ag/CuO nanocomposites produced from Murraya koenigii and Zingiber officinale was assessed in vitro, utilising glucose-6-phosphatase, α-glucosidase, and α-amylase tests. CuONPs, AgONPs, and Ag/CuO composites have been synthesised utilising a chemical approach for comparison. The findings indicated that the Ag/CuO composite synthesised using photosynthesis exhibited the most significant promise for treating diabetes compared to the other materials examined, and it can be attributed to the presence of a more substantial amount of phytoconstituents in these extracts.^207^

### 4.10 Dental application of copper oxide nanoparticles

In dentistry, metal nanoparticles are used because of their distinct features that rely on their shape. These qualities include their various nanosizes and shapes, enormous surface-area-to-volume ratio, and unique distribution. The NPs possess properties that improve their antibacterial efficacy, bio-physio-chemical functionalization, and biocompatibility.

CuONPs are used to enhance the chemical and physical properties of various dental materials, including tooth amalgam material, obturation materials, adhesives, implants for teeth, endodontic-irrigation solutions, resins, as well as orthodontic brackets and archwires.^208^ Detachable and permanent incomplete denture framework designs that include copper NPs can effectively treat mouth infections and denture-induced stomatitis in dentistry.^209^ A study revealed the existence of a titanium alloy implant containing copper that exhibits anti-infective properties, making it potentially effective against oral bacteria. The research demonstrated that the titanium-copper alloy effectively combated peri-implant infections and showed favourable biocompatibility.^210^ Prior research studies have documented that the titanium-copper alloy exhibits properties that counteract the effects of ageing and inhibit the growth of microorganisms. The research showed that the antibacterial properties could be altered by manipulating the copper concentration in the alloy composition.^211^ A study found that copper-containing mesoporous bio-glass reduced microbial activity and biofilm development, attributed to the release of copper ions.^212^ Regarding orthodontic appliances, including copper nanoparticles in a nickel-titanium alloy offers several advantages. The inclusion of CuONPs in the archwire resulted in a reduction in loading stress and an increase in unloading stress.^213^

Due to its affordability and low toxicity, copper is an ideal metal for use as a catalyst. Additionally, copper-based catalysts can be easily recycled and reused, as stated in reference.

### 4.11 Biocatalyst and bioremediation

Bioremediation is crucial for treating polluted water contaminated with substances like dyes, as it can cause disruptions in aquatic ecosystems and pose significant risks.^214^ The qualities of CuONPs are examined to remedy contaminated water bodies caused by textile effluents, industrial discharge, and similar sources. CuONPs have been found effective in purifying wastewater.^215^ Additionally, the catalytic capability of CuONPs has been seen in lowering the activity of Xanthene dye, along with potent reducing agents. These reducing agents act as precursors of Cu2+ ions, which then contribute to the synthesis of CuONPs. This process ultimately leads to the quenching of fluorescence. These concepts could also be relevant in biological sensors and bio-labelling.^216^ The degradation activity of CuONPs was higher than that of Ni@Fe3O4 nanoparticles when tested against organic dyes such as the colour methylene blue and Rhodamine B.^217^

Additionally, CuONPs demonstrated the ability to reduce 4-nitrophenol,^218^ making them suitable for treating textile wastewater. Aspergillus species are known to produce aflatoxins (AFs), which have been reported to be carcinogenic and mutagenic. A study discovered that the adsorbent capacity of CuONPs for aflatoxin B1, or AF, was greater than that of Ag-NPs but lower than that of Fe-NPs.^219^

### 4.12 Used in plant growth

Various parameters, such as the concentration, size, plant species, and particle structure, influence the plant growth responses mediated by CuONP. A study revealed that CuONPs could enhance the growth of roots and shoots in *Triticum aestivum* and Phaseolus radiate. The utilisation of CuONPs resulted in enhanced development of roots and shoots in Zea mays.^220^ When Allium cepa was treated with CuONPs at a dosage of 20 μg/mL, there was an improvement in growth and an increase in the mitotic index. The mitotic index declined as the amounts of CuONPs increased.^221^ When CuONPs were applied at a higher dosage to *Arabidopsis thaliana* seedlings, it resulted in the Inhibition of both root and shoot growth, as well as a decrease in chlorophyll levels.^222^

Conversely, an elevated concentration of CuONPs adversely affects plant growth. The consumption of copper nanoparticles at concentrations that range from 200 to 1000 mg/l adversely affects the development and growth of *Triticum aestivum*, Raphanus sativus, Phaseolus radiates, and Lolium perenne.^223^ The growth response typically depends on the concentration of CuONPs, and applying copper nanoparticles at doses of 20, 25, 30, and 35 ppm improved wheat plants' growth and yields. Concentrations over 1000 parts per million (ppm) resulted in a decline in wheat growth, leading to a fall in yields.^224^ The utilisation of CuONPs on transgenic cotton plants increased the expression of foreign genes responsible for producing Bt toxins in the leaves.^225^ Our recent research has demonstrated that applying CuONPs dramatically enhances the activity of defence enzymes, total phenol levels, and other defence parameters in Lens culinaris plants. This effect is achieved by activating the nitric oxide signalling pathway.^226^

### 4.13 Photocatalytic activity of copper oxide nanoparticles

Studies have shown that metal and nanoparticles made of metal oxide demonstrate high photocatalytic effectiveness.^227^^,^^228^ Researchers have found that employing CuONPs synthesised with Thymus vulgaris leaf extract is a highly efficient accelerator for the N-arylation of amines and indoles, resulting in the substantial production of N-arylated products. The catalyst for supplementary catalytic processes was successfully recovered and recycled without any loss in activity.^229^ The evaluation of the photocatalytic degradation of CuONPs, synthesised using green methods, on RB dye showed a degradation efficiency of 94 % up to the fifth cycle, which indicates that the photosynthesised CuONPs are durable and practical as a photocatalytic agent.^230^ In addition, the comparison of the catalytic properties of copper oxide (CuONPs) and zinc oxide nanoparticles (ZnONPs) for the breakdown of simple violet 3 demonstrated that ZnONPs had greater catalytic efficiency than CuONPs.^65^ The investigation of CuONPs, created from aloe plant leaves and facilitated by solar simulator light, demonstrated complete degradation of methylene blue within a 10-min timeframe. The high activity level can be attributed to the phytochemicals present in the leaves of Aloe Vera.^231^

### 4.14 Role of CuO nanoparticles in tissue engineering

#### 4.14.1 Scaffold materials

CuO nanoparticles can enhance the properties of scaffold materials used in tissue engineering by improving mechanical strength and promoting cell growth. Incorporating CuO nanoparticles into polymeric or ceramic scaffolds can enhance their mechanical properties and provide bioactive cues to cells.^232^ A recent study developed polycaprolactone (PCL) scaffolds embedded with CuO nanoparticles for bone tissue engineering. The scaffolds showed enhanced mechanical strength and osteoinductive properties, promoting bone regeneration in rat femur defect models.^233^

#### 4.14.2 Regenerative medicine

CuO nanoparticles can stimulate the differentiation of stem cells into various tissue-specific cells, aiding in tissue regeneration. CuO nanoparticles were used to enhance the regenerative capacity of chitosan-based hydrogels for wound healing.^234^ The composite hydrogels showed accelerated wound closure and improved epithelialization in diabetic rat models, highlighting their potential in regenerative medicine.^235^

## 5 Toxicological assessment of copper oxide nanoparticles

Nanotechnology and its applications have led to more exposure of Nanoparticles to the atmosphere and humans in recent decades. The presence of NPs in the environment has an enormous effect on the health of humans.^236^, ^237^, ^238^, ^239^ Research on MONPs' toxicity has primarily focused on causing cytotoxicity in biological systems. Dissolution of metallic ions from Nanoparticles and the atmosphere in which they are delivered are crucial factors in generating toxicity.^240^

**Fig. 5:**
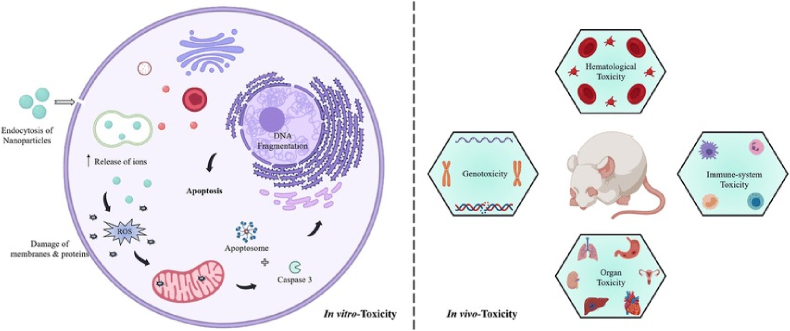
Schematic illustration of in vitro and in vivo systems implemented for toxicity assessment of CuONPs.

Physiological analysis, in vitro studies, and in vivo research are the three main approaches to assessing nanomaterials' toxic effects.^241^ Among several techniques, the in vivo test is considered morally sound, cost-effective, highly valid and reliable in assessing risks. Conducting in vitro studies and analysing short-term toxicity can significantly enhance the understanding of mechanisms in nanotoxicology (Fig. 5).^242^ Due to conflicting toxicological findings, more in vitro and in vivo investigations are needed to determine the determinants and processes of nanomaterial-mediated toxicity. The critical problem is standardising and regulating investigative methodologies and creating a database of NP dangers for investigators, producers, and consumers to access. Less information is available regarding the toxicity assessment of natural MONPs using in vivo study and in vitro analysis methodologies compared to other manufacturing techniques. This section covers the in vitro and in vivo toxicity of CuO NPs, regardless of the synthesis technique.

### 5.1 In vitro toxicity of copper oxide nanoparticles

The recent focus on CuO NP toxicity is significant. This section stresses CuO NPs' in vitro oxidative stress toxicity. They have investigated the dose-dependent toxicity of NPs made from CuO in Hep-2 pulmonary airway epithelial cells in vitro. Results show HEP-cell appearance alterations following 24 h of exposure to 1–40 μg/mL CuO NPs. Oxidative stress caused cytotoxicity, which increased LPO and ROS and decreased GSH and matrix metalloproteinase. CuONPs were tested for toxicity in a gastrointestinal model^243^ and lung and human melanoma cells.^244^ In Hep-2 cells, changed cell death genes and activated caspase enzymes caused programmed cell death.^245^ Recent research examined the toxicity of novel compounds on human embryonic kidney cell lines. Euphorbia heterophylla was used to synthesise eco-friendly CuONPs for NC films. NC films increased cell viability by almost 80 %.^246^ CuONPs made from Rhus punjabensis leaf extract were harmful to brine shrimp Leishmania tropica in vitro.^247^ They were testing CuO NPs' in vitro cytotoxic and oxidative stress in A549 and HepG2 cells. HepG2 cells, on the other hand, had lower glutathione levels than A549 cells, which had more lipid peroxidation and ROS generation.

Malondialdehyde (MDA), a marker of lipid peroxidation, showed a rise, while antioxidant enzymes such as SOD and CAT demonstrated an increase. Additionally, the level of glutathione (GSH) decreased. According to the findings, oxidative stress may be the leading cause of CuO NP toxicity. CuONPs can potentially cause injury to the HepG2 cells and liver cells of catfish by generating reactive oxygen species (ROS).^248^^,^^249^ The effects of PbO and CuONPs on human fibroblasts were evaluated. They assessed a substance's cytotoxicity by measuring its impact on cellular dehydrogenase activity and ATP content in vitro. They have measured cell proliferation, viability, substrate adhesion, and spreading using continuous impedance-based normalised cell index measurement. The parameters showed that CuO and PbO NPs damaged human fibroblasts depending on dosage.^250^ CuONPs exhibited cytotoxic effects on various human cell types, including lung epithelial A549 cells, cardiovascular endothelial cells, renal cells, and nerve cells. Another study reported no alteration in SOD activity but observed a 25 % and 29 % reduction in CAT and GR activity, respectively.^251^ Researchers found that 80 lg/cm2 CuO NPs increased 8-isoprostanes by 100 % and GPx activity by 150 % in Hep-2 cells. Oxidised GSH prevented epithelial cells from suppressing CuO NP-generated ROS, as the oxidised to total glutathione ratio increased by 150 %. Cell damage and death are caused by oxidative stress. CuONPs resulted in lesions and damage in A549 cells, leading to cytotoxicity. This cytotoxic effect was observed to depend on both the exposure duration and the concentration of CuONPs.^252^ CuO and PbO NPs had similar lethal effects in a not specific in vivo toxicity investigation.^253^ A study examined the in vitro cytotoxicity of chemically and greenly synthesised CuO NPs. The CuONPs displayed significant toxicity, which was dose-dependent. This toxicity was attributed to the generation of ROS, leading to apoptosis and necrosis. Following the therapy, lymphocytes exhibited hemolysis, decreased endurance, elevated intracellular accumulation, generation of NO, NADPH oxidase activity, TNF-α, MDA, LDH, and pro-apoptotic enzymes. Nevertheless, the levels of interleukin and antiapoptotic proteins exhibited a reduction.^67^ A further investigation revealed that the application of Monotheca buxifolia CuONPs resulted in dose-dependent mortality of brine shrimp, with deadly effects observed at 200, 100, 50, and 25 μg/mL dosages. At a dosage level of 200 μg/mL, the maximum fatality rate reported was 80 ± 0.970, while lower doses led to a reduction in mortality of 40 ± 0.7. A 40.3 μg/mL value was determined using Table Curve two-dimensional v5.01 software.^254^ When CuO nanoparticles (measuring 50 nm) were introduced to respiratory epithelial cells, the level of DNA damage varied depending on the concentration. Mortality was induced by the peroxidation of lipids and the resulting oxidative stress.^255^ HepG2 cells treated with 22 nm CuONPs demonstrated elevated generation of ROS, enhanced toxicity to cells, increased expression of p53 and caspase-3, and initiation of mitochondrial cell death.^256^ A human pulmonary epithelium cell (BEAS-2B) was adversely affected by CuONPs measuring 20–200 nm. NPs damage cells through oxidative stress, cell cycle disruption, and planned cell death. This study shows that NPs cause oxidative stress and genetic harm to cells.^257^

### 5.2 In vivo toxicity of copper oxide nanoparticles

The immunotoxic and antioxidant effects of CuONPs were assessed on Six-week-old female mice over six weeks. The pulmonary system and liver accumulated copper more than other organs. There was also a considerable rise in IL-4, IL-5, IL-12p70 and IFN-g production and splenocyte and T-lymphocyte proliferation. Immunogenic experiments showed granulocytes had excellent phagocytic activity with a decreased respiratory burst, while monocytes did not differ. The thymus, spleen, and lymph nodes had similar haematological characteristics and cell subset proportions. In addition, CuONPs-treated animals had significantly lower GSH levels, indicating antioxidant status alterations. The data suggest CuONPs produce undesirable immune response fluctuations.^258^ After two weeks of oral CuONPs, rats' memory and learning were somewhat altered. CuONPs affect rats' locomotor activity, causing increased anxiety and liver/stomach weights but causing modest metabolic changes.^259^ The study investigated the detrimental effects of biologically synthesised copper oxide nanoparticles (B–CuO) and commercially synthesised copper oxide nanoparticles (C–CuO) on mice. The nanoparticles were given orally at 500 mg/kg of body weight. Both B–CuO and C–CuO nanoparticles caused an increase in white blood cell count, raised levels of ATL, AST, creatinine and urea in the blood and liver tissues, and increased the production of P53 mRNA and caspase-3 protein. In addition, CuONPs cause inflammation and degradation in the kidneys, liver, and splenic tissues. Upon analysing both types of nanoparticles (NPs), it was found that B–CuO NPs exhibited a higher degree of toxicity compared to C–CuO NPs.^251^ They have analysed the harmful effects of ZnO and CuONPs on Cornu aspersum (land snails), uncovering the clumping together of the nanoparticles in the snails' blood cells. As a result, there was an elevation in reactive oxygen species (ROS), oxidation of lipids, carbonyl protein concentration, ubiquitin conjugates, and degradation of caspase conjugate levels. ZnO nanoparticles exhibited more pronounced toxic effects compared to CuONPs.^260^ Exposing zebrafish embryos to CuO NPs derived from plants caused the accumulation of these particles on the skin surfaces and chorion. This buildup caused problems with the embryonic sacs and the growth of pericardial swelling.^261^

Researchers looked at how dangerous CuONPs/ascorbate (ASC) and CuO NPs/polyethyleneimine (PEI) were to rats' lungs over five days using a dose-dependent method. Six and twenty-seven days after contact, both the CuO NPs/ASC and CuO NPs/PEI types showed changes in the lung. In the case of CuO NPs/ASC, rats exhibited dysregulation in genes associated with drug metabolism.^262^ Neurotoxicity of CuONPs was assessed in a group of A total of 30 Wister albino mice were observed for 28 days. Pomegranate juice (PJ) contains high levels of polyphenols, a class of chemicals renowned for their antioxidant properties. This study investigated the molecular role of PJ in mitigating the toxicity of CuONPs. Elevated MDA levels and reduced antioxidant capability were observed in all brain areas like the cerebrum, cerebellum and hippocampus. Rats exposed to CuONPs without PJ experienced memory impairment as well as cognitive and mental issues.

Prolonged exposure to nanoparticles resulted in elevated levels of caspase-3, iNOS, and GFAP while causing a decrease in the transcript levels of HO-1/Nrf2 proteins in brain tissues. On the other hand, rats that received PJ treatment demonstrated enhancements in all neurotoxicological assessments. In summary, PJ decreased oxidative stress by increasing the expression of HO-1/Nrf2 transcripts.^263^ The synthesised CuONPs exhibited neurotoxicity in female and male rats; dependent on the dosage and duration of exposure, renal functional tests (RFTs), lipid profiles, and histopathology remained steady. Nevertheless, the males who received a large dosage exhibited notable toxicity. CuONPs exhibited a reasonably high level of biocompatibility at lower doses.^33^ A study discovered that artificially produced CuONPs, when administered in higher amounts, caused liver toxicity in both the parents and their children. This conclusion was backed by liver function tests, examination of tissue samples, studies on genetic damage, and analysis of antioxidant enzyme activity.^264^ A separate study has demonstrated that long-term inhalation of CuONPs leads to lung inflammation. In bronchoalveolar lavage fluid, the enzyme lactate dehydrogenase, macrophages, total cell counts, inflammatory substances, neutrophils, iron, and lung weight increased.

Additionally, lung dosimetry and BALF measurements showed a constant rise in the copper (Cu) concentration after delivery and a decrease following exposure. Cu concentrations in the blood and heart increased significantly, suggesting Cu translocation into the bloodstream and heart tissue. Copper (Cu) removal from the respiratory tract has a 6.5-day half-life. Cu also caused the renal system to gain weight and the spleen to shrink, indicating its harmful effects. Selenium levels dropped, indicating a slight element homeostasis imbalance.^265^

## 6 Comparative analysis with other nanoparticles

Nanotechnology has revolutionized the field of biomedicine, introducing a variety of nanoparticles with unique properties and applications. Among the most widely studied metal oxide nanoparticles are copper oxide (CuO), zinc oxide (ZnO), and titanium dioxide (TiO2). Each of these nanoparticles exhibits distinct chemical and physical characteristics that influence their performance in biomedical applications. Copper oxide nanoparticles (CuO NPs) are renowned for their potent antimicrobial activity and potential in cancer therapy due to their ability to generate reactive oxygen species (ROS).^266^ This ROS generation, while beneficial for certain therapeutic applications, also raises concerns regarding cytotoxicity and biocompatibility. Despite these challenges, advancements in surface modification and functionalization have enhanced the safety and efficacy of CuO NPs.^267^

## 7 Challenges and future prospects

The use of copper nanoparticles (CuONPs) in various applications, particularly in biomedicine and environmental technologies, has seen significant growth. However, several challenges persist that impede their widespread adoption and efficiency. Addressing these challenges while preserving their bioactivity is crucial for their future development and application.

### 7.1 Morphology and surface characteristics

The morphology and surface characteristics of CuONPs play a critical role in determining their chemical reactivity, stability, and interaction with biological systems. Variations in particle size, shape, and surface roughness can significantly influence the efficacy of CuONPs. Irregular morphologies may lead to inconsistent performance, while smooth, well-defined shapes can enhance uniformity in applications such as catalysis and drug delivery.^268^ The tendency of CuONPs to agglomerate due to their high surface energy is another issue, leading to reduced surface area and loss of unique properties. Innovative surface modification techniques, such as coating with polymers or surfactants, can mitigate these issues by stabilising the nanoparticles and preventing agglomeration.^269^

### 7.2 Hydrophobic and porous variations

Hydrophobic and porous copper nanoparticles present additional challenges and opportunities. Hydrophobic CuONPs are advantageous in applications where water resistance is necessary, such as in antimicrobial coatings. However, achieving uniform hydrophobicity while maintaining bioactivity can be difficult. Porous CuONPs, on the other hand, offer a high surface area to volume ratio, beneficial for catalysis and environmental remediation. Ensuring that these porous structures maintain their integrity and do not collapse or degrade over time is crucial. Advanced fabrication methods, including templating and controlled synthesis processes, are being explored to create robust hydrophobic and porous CuONPs that retain their functional properties over extended periods.

### 7.3 Innovative strategies to address challenges

To preserve the bioactivity of CuONPs while overcoming these morphological and surface characteristic challenges, several innovative strategies have been proposed.

1. Surface Functionalization: Coating CuONPs with biocompatible materials such as polyethylene glycol (PEG) or natural polymers like chitosan can enhance their stability and reduce toxicity. These coatings can also provide specific functional groups that facilitate targeted delivery in biomedical applications.
2. Core-Shell Structures: Developing core-shell nanoparticles, where CuONPs are encapsulated within another material (e.g., silica, gold), can protect the core from oxidation and agglomeration, thereby preserving its bioactive properties.
3. Green Synthesis Methods: Utilising plant extracts or microbial processes for synthesising CuONPs can produce more stable and biocompatible nanoparticles. These green synthesis methods often result in nanoparticles with better-controlled size and shape, enhancing their application potential.
4. Controlled Release Systems: Embedding CuONPs in hydrogels or other controlled-release matrices can help in maintaining a steady release rate of copper ions, crucial for applications such as wound healing and antimicrobial treatments.^270^

### 7.4 Public perception

Public perception of CuONPs is a critical factor that influences their acceptance and application. Concerns regarding the potential toxicity and environmental impact of nanoparticles can hinder their adoption. Unlike gold and silver nanoparticles, which have a longer history of use and are often perceived as more benign, copper nanoparticles face skepticism due to their reactivity and potential for generating reactive oxygen species (ROS). Effective communication of the safety measures, environmental benefits, and regulatory compliance associated with CuONPs is essential to improve public perception.

### 7.5 Scientific community interest

Despite the unique advantages of CuONPs, the scientific community often shows a preference for using gold and silver nanoparticles. This preference is due to the well-documented synthesis protocols, stability, and biocompatibility of gold and silver nanoparticles.^271^ However, the cost-effectiveness and abundant availability of copper make CuONPs a highly attractive alternative, particularly for large-scale applications.^272^ There is a growing interest in the scientific community to explore the potential of CuONPs, driven by advancements in synthesis and functionalization techniques that address their inherent challenges. Research is increasingly focusing on leveraging the distinctive properties of CuONPs while ensuring their safe and sustainable use.^273^

### 7.6 Future prospects

The future of CuONPs lies in interdisciplinary research that bridges materials science, nanotechnology, and biology. Innovations in nanoparticle synthesis, coupled with a better understanding of their interactions with biological systems, will pave the way for new applications in healthcare, environmental protection, and beyond. Collaborative efforts between academia, industry, and regulatory bodies will be crucial in developing standards and guidelines that ensure the safe and effective use of copper nanoparticles. As the scientific community continues to uncover the full potential of CuONPs, addressing the associated challenges will be key to their successful integration into a wide array of technological advancements.

## 8 Conclusion

The advancement in the environmentally friendly production of nanoparticles has brought an entirely novel perspective to biomedical applications. CuONPs synthesised from plants possess numerous beneficial properties, including antimicrobial, antiviral, anti-inflammatory, anticancer, and wound healing effects. It's crucial to highlight that copper oxide nanoparticles produced from various plants exhibit varied applications because of variations in metabolite composition. The diverse array of attributes gives rise to variations in the sizes, arrangement, and composition of the NPs, causing alterations in their overall attributes and functionalities. Additionally, copper oxide nanoparticle processes and pathways must be verified at the cellular and organismic levels. Hence, conducting a thorough examination of these nanoparticles, encompassing both in vivo and in vitro evaluations from various angles, could help determine their medicinal use. Despite the need for further trials and research, the future looks promising for developing copper and copper oxide nanoparticles as potential drugs, particularly in cancer therapy. Utilising natural plant resources for green technology in nanobiotechnology is on the brink of a breakthrough.

## Funding

No funds are available for this article.

## Authors’ contributions

All authors contributed in the manuscript. All authors read and approved the manuscript.

## Declaration of competing interest

The authors declare that they have no known competing financial interests or personal relationships that could have appeared to influence the work reported in this paper.

## References

[1] Bezza F.A., Tichapondwa S.M., Chirwa E.M.N. (2020). Fabrication of monodispersed copper oxide nanoparticles with potential application as antimicrobial agents. Sci Rep.

[2] Folorunso A., Akintelu S., Oyebamiji A.K. (2019). Biosynthesis, characterization and antimicrobial activity of gold nanoparticles from leaf extracts of Annona muricata. J Nanostructure Chem.

[3] Akintelu S.A., Folorunso A.S., Ademosun O.T. (2019). Instrumental characterization and antibacterial investigation of silver nanoparticles synthesized from Garcinia kola leaf. J Drug Deliv Therapeut.

[4] Adewale S.A., Similoluwa A.F. (2019). Biosynthesis, characterization and antifungal investigation of Ag-Cu nanoparticles from bark extracts of Garcina kola. sciencepub.

[5] Oves M., Ahmar Rauf M., Aslam M. (2022). Green synthesis of silver nanoparticles by Conocarpus Lancifolius plant extract and their antimicrobial and anticancer activities. Saudi J Biol Sci.

[6] Javed R., Ain N ul, Gul A. (2022). Diverse biotechnological applications of multifunctional titanium dioxide nanoparticles: an up-to-date review. IET Nanobiotechnol.

[7] Katwal R., Kaur H., Sharma G., Naushad M., Pathania D. (2015). Electrochemical synthesized copper oxide nanoparticles for enhanced photocatalytic and antimicrobial activity. J Ind Eng Chem.

[8] Grigore M.E., Biscu E.R., Holban A.M., Gestal M.C., Grumezescu A.M. (2016). Methods of synthesis, properties and biomedical applications of CuO nanoparticles. Pharmaceuticals.

[9] Bhanushali S., Ghosh P., Ganesh A., Cheng W. (2015).

[10] Naz S., Gul A., Zia M. (2020). Toxicity of copper oxide nanoparticles: a review study. IET Nanobiotechnol.

[11] Dang T.M.D., Le T.T.T., Fribourg-Blanc E., Dang M.C. (2011). Synthesis and optical properties of copper nanoparticles prepared by a chemical reduction method. Adv Nat Sci Nanosci Nanotechnol.

[12] Ealias A.M., Saravanakumar M.P. (2017). IOP Conference Series: Materials Science and Engineering.

[13] Sharad Kamble Mr, Kaveri Bhosale Miss, Mohite Mr Mahesh, Navale Mrs Swapnali (2023). Methods of preparation of nanoparticles. Int. J. Adv. Res. Comput. Sci..

[14] Sangeetha S., Kalaignan G.P., Anthuvan J.T. (2015). Pulse electrodeposition of self-lubricating Ni-W/PTFE nanocomposite coatings on mild steel surface. Appl Surf Sci.

[15] Pandiyarajan T., Udayabhaskar R., Vignesh S., James R.A., Karthikeyan B. (2013). Synthesis and concentration dependent antibacterial activities of CuO nanoflakes. Mater Sci Eng C.

[16] Yahia I.S., Farag A.A.M., El-Faify S., Yakuphanoglu F., Al-Ghamdi A.A. (2016). Synthesis, optical constants, optical dispersion parameters of CuO nanorods. Optik.

[17] Sahooli M., Sabbaghi S., Saboori R. (2012). Synthesis and characterization of mono sized CuO nanoparticles. Mater Lett.

[18] Mohamed R.M., Harraz F.A., Shawky A. (2014). CuO nanobelts synthesized by a template-free hydrothermal approach with optical and magnetic characteristics. Ceram Int.

[19] Hasan S.S., Singh S., Parikh R.Y. (2008). Bacterial synthesis of copper/copper oxide nanoparticles. J Nanosci Nanotechnol.

[20] Jiang T., Wang Y., Meng D., Yu M. (2015). Facile synthesis and photocatalytic performance of self-assembly CuO microspheres. Superlattice Microst.

[21] Safarifard V., Morsali A. (2012). Sonochemical syntheses of a nano-sized copper(II) supramolecule as a precursor for the synthesis of copper(II) oxide nanoparticles. Ultrason Sonochem.

[22] Karthik A.D., Geetha K. (2013). Synthesis of copper precursor, copper and its oxide nanoparticles by green chemical reduction method and its antimicrobial activity. J Appl Pharmaceut Sci.

[23] Zhu H.T., Lin Y.S., Yin Y.S. (2004). A novel one-step chemical method for preparation of copper nanofluids. J Colloid Interface Sci.

[24] Liu Q.M., Zhou D.B., Yamamoto Y.Y., Kuruda K., Okido M. (2012). Effects of reaction parameters on preparation of Cu nanoparticles via aqueous solution reduction method with NaBH4. Trans Nonferrous Metals Soc China.

[25] Su X., Zhao J., Bala H. (2007). Fast synthesis of stable cubic copper nanocages in the aqueous phase. J Phys Chem C.

[26] Vijay Kumar P.P.N., Pammi S.V.N., Kollu P., Satyanarayana K.V.V., Shameem U. (2014). Green synthesis and characterization of silver nanoparticles using Boerhaavia diffusa plant extract and their anti bacterial activity. Ind Crops Prod.

[27] Khan I., Saeed K., Khan I. (2019). Nanoparticles: properties, applications and toxicities. Arab J Chem.

[28] Mandal D., Bolander M.E., Mukhopadhyay D., Sarkar G., Mukherjee P. (2006). The use of microorganisms for the formation of metal nanoparticles and their application. Appl Microbiol Biotechnol.

[29] Kowshik M., Ashtaputre S., Kharrazi S. (2003). Extracellular synthesis of silver nanoparticles by a silver-tolerant yeast strain MKY3. Nanotechnology.

[30] Mukherjee P., Ahmad A., Mandal D. (2001). Fungus-mediated synthesis of silver nanoparticles and their Immobilization in the mycelial matrix: a novel biological approach to nanoparticle synthesis. Nano Lett.

[31] Chaudhary R., Nawaz K., Khan A.K., Hano C., Abbasi B.H., Anjum S. (2020). An overview of the algae‐mediated biosynthesis of nanoparticles and their biomedical applications. Biomolecules.

[32] Iravani S. (2011). Green synthesis of metal nanoparticles using plants. Green Chem.

[33] Naz S., Gul A., Zia M., Javed R. (2023). Synthesis, biomedical applications, and toxicity of CuO nanoparticles. Appl Microbiol Biotechnol.

[34] Chevallet M., Veronesi G., Fuchs A., Mintz E., Michaud-Soret I., Deniaud A. (2017). Impact of labile metal nanoparticles on cellular homeostasis. Current developments in imaging, synthesis and applications. Biochim Biophys Acta Gen Subj.

[35] Naz S., Shahzad H., Ali A., Zia M. (2018). Nanomaterials as nanocarriers: a critical assessment why these are multi-chore vanquisher in breast cancer treatment. Artif Cells, Nanomed Biotechnol.

[36] Katsumiti A., Thorley A.J., Arostegui I. (2018). Cytotoxicity and cellular mechanisms of toxicity of CuO NPs in mussel cells in vitro and comparative sensitivity with human cells. Toxicol Vitro.

[37] Abdulateef S.A., Matjafri M.Z., Omar A.F. (2016). AIP Conference Proceedings..

[38] Yang B., Chen D. (2017). Synthesis of CuO nanoparticles for catalytic application via ultrasound-assisted ball milling. Processing and Application of Ceramics.

[39] Baláž M., Tešinský M., Marquardt J. (2020). Synthesis of copper nanoparticles from refractory sulfides using a semi-industrial mechanochemical approach. Adv Powder Technol.

[40] Das A., Kushwaha A., Bansal N.R. (2016). Copper oxide nano-particles film on glass by using sputter and chemical bath deposition technique. Adv Mater Lett.

[41] Amendola V., Meneghetti M. (2013). What controls the composition and the structure of nanomaterials generated by laser ablation in liquid solution?. Phys Chem Chem Phys.

[42] Dörner L., Cancellieri C., Rheingans B. (2019). Cost-effective sol-gel synthesis of porous CuO nanoparticle aggregates with tunable specific surface area. Sci Rep.

[43] Arunkumar B., Johnson Jeyakumar S., Jothibas M. (2019). A sol-gel approach to the synthesis of CuO nanoparticles using Lantana camara leaf extract and their photo catalytic activity. Optik.

[44] Odularu A.T. (2018). Metal nanoparticles: thermal decomposition, biomedicinal applications to cancer treatment, and future perspectives. Bioinorgan Chem Appl.

[45] Effenberger F.B., Sulca M.A., Machini M.T. (2014). Copper nanoparticles synthesized by thermal decomposition in liquid phase: the influence of capping ligands on the synthesis and bactericidal activity. J Nanoparticle Res.

[46] Phiwdang K., Suphankij S., Mekprasart W., Pecharapa W. (2013). Synthesis of CuO nanoparticles by precipitation method using different precursors. Energy Proc.

[47] Gvozdenko A.A., Siddiqui S.A., Blinov A.V. (2022). Synthesis of CuO nanoparticles stabilized with gelatin for potential use in food packaging applications. Sci Rep.

[48] Javed R., Rais F., Kaleem M. (2021). Chitosan capping of CuO nanoparticles: Facile chemical preparation, biological analysis, and applications in dentistry. Int J Biol Macromol.

[49] Rostami-Tapeh-Esmaeil E., Golshan M., Salami-Kalajahi M., Roghani-Mamaqani H. (2021). Synthesis of copper and copper oxide nanoparticles with different morphologies using aniline as reducing agent. Solid State Commun.

[50] Silva N., Ramírez S., Díaz I., Garcia A., Hassan N. (2019). Easy, quick, and reproducible sonochemical synthesis of CuO nanoparticles. Materials.

[51] Nasrollahzadeh M., Sajjadi M., Dadashi J., Ghafuri H. (2020). Pd-based nanoparticles: plant-assisted biosynthesis, characterization, mechanism, stability, catalytic and antimicrobial activities. Adv Colloid Interface Sci.

[52] Nabila M.I., Kannabiran K. (2018). Biosynthesis, characterization and antibacterial activity of copper oxide nanoparticles (CuO NPs) from actinomycetes. Biocatal Agric Biotechnol.

[53] Jadoun S., Arif R., Jangid N.K., Meena R.K. (2021). Green synthesis of nanoparticles using plant extracts: a review. Environ Chem Lett.

[54] Ghidan A.Y., Al-Antary T.M., Awwad A.M. (2016). Green synthesis of copper oxide nanoparticles using Punica granatum peels extract: effect on green peach Aphid. Environ Nanotechnol Monit Manag.

[55] Gunalan S., Sivaraj R., Venckatesh R. (2012). Aloe barbadensis Miller mediated green synthesis of mono-disperse copper oxide nanoparticles: optical properties. Spectrochim Acta Mol Biomol Spectrosc.

[56] Prakash S., Elavarasan N., Venkatesan A., Subashini K., Sowndharya M., Sujatha V. (2018). Green synthesis of copper oxide nanoparticles and its effective applications in Biginelli reaction, BTB photodegradation and antibacterial activity. Adv Powder Technol.

[57] Kumar P.P.N.V., Shameem U., Kollu P., Kalyani R.L., Pammi S.V.N. (2015). Green synthesis of copper oxide nanoparticles using aloe vera leaf extract and its antibacterial activity against fish bacterial pathogens. Bionanoscience.

[58] Ijaz F., Shahid S., Khan S.A., Ahmad W., Zaman S. (2017). Green synthesis of copper oxide nanoparticles using abutilon indicum leaf extract: antimicrobial, antioxidant and photocatalytic dye degradation activities. Trop J Pharmaceut Res.

[59] Sutradhar P., Saha M., Maiti D. (2014). Microwave synthesis of copper oxide nanoparticles using tea leaf and coffee powder extracts and its antibacterial activity. J Nanostructure Chem.

[60] Devi H.S., Singh T.D. (2014). Synthesis of copper oxide nanoparticles by a novel method and its application in the degradation of Methyl orange. Adv Electron Elec Eng.

[61] Asemani M., Anarjan N. (2019). Green synthesis of copper oxide nanoparticles using Juglans regia leaf extract and assessment of their physico-chemical and biological properties. Green Process Synth.

[62] Jayakumarai G., Gokulpriya C., Sudhapriya R., Sharmila G., Muthukumaran C. (2015). Phytofabrication and characterization of monodisperse copper oxide nanoparticles using Albizia lebbeck leaf extract. Appl Nanosci.

[63] Fardood S.T., Ramazani A. (2016). Green synthesis and characterization of copper oxide nanoparticles using coffee powder extract. J Nanostruct.

[64] Yedurkar S.M., Maurya C.B., Mahanwar P.A. (2017). A biological approach for the synthesis of copper oxide nanoparticles by ixora coccinea leaf extract. J Mater Environ Sci.

[65] Sorbiun M., Shayegan Mehr E., Ramazani A., Taghavi Fardood S. (2018). Green synthesis of zinc oxide and copper oxide nanoparticles using aqueous extract of Oak fruit Hull (jaft) and comparing their photocatalytic degradation of basic violet 3. Int J Environ Res.

[66] Sivaraj R., Rahman P.K.S.M., Rajiv P., Narendhran S., Venckatesh R. (2014). Biosynthesis and characterization of Acalypha indica mediated copper oxide nanoparticles and evaluation of its antimicrobial and anticancer activity. Spectrochim Acta Mol Biomol Spectrosc.

[67] Dey A., Manna S., Chattopadhyay S. (2019). Azadirachta indica leaves mediated green synthesized copper oxide nanoparticles induce apoptosis through activation of TNF-α and caspases signaling pathway against cancer cells. J Saudi Chem Soc.

[68] Narasaiah P., Mandal B.K., Sarada N.C. (2017). IOP Conference Series: Materials Science and Engineering.

[69] Chand Mali S., Raj S., Trivedi R. (2019). Biosynthesis of copper oxide nanoparticles using Enicostemma axillare (Lam.) leaf extract. Biochem Biophys Rep.

[70] Nagajyothi P.C., Muthuraman P., Sreekanth T.V.M., Kim D.H., Shim J. (2017). Green synthesis: in-vitro anticancer activity of copper oxide nanoparticles against human cervical carcinoma cells. Arab J Chem.

[71] Rehana D., Mahendiran D., Kumar R.S., Rahiman A.K. (2017). Evaluation of antioxidant and anticancer activity of copper oxide nanoparticles synthesized using medicinally important plant extracts. Biomed Pharmacother.

[72] Kiriyanthan R.M., Sharmili S.A., Balaji R. (2020). Photocatalytic, antiproliferative and antimicrobial properties of copper nanoparticles synthesized using Manilkara zapota leaf extract: a photodynamic approach. Photodiagnosis Photodyn Ther.

[73] Mandava K., Kadimcharla K., Keesara N.R., Fatima S.N., Bommena P., Batchu U.R. (2017). Green synthesis of stable copper nanoparticles and synergistic activity with antibiotics. Indian J Pharmaceut Sci.

[74] Ali K., Saquib Q., Ahmed B. (2020). Bio-functionalized CuO nanoparticles induced apoptotic activities in human breast carcinoma cells and toxicity against Aspergillus flavus: an in vitro approach. Process Biochem.

[75] Taghavi Fardood S., Ramazani A., Asiabi P.A., Joo S.W. (2018). A novel green synthesis of copper oxide nanoparticles using a henna extract powder. J Struct Chem.

[76] Akhter S.M.H., Mohammad F., Ahmad S. (2019). Terminalia belerica mediated green synthesis of nanoparticles of copper, iron and zinc metal oxides as the alternate antibacterial agents against some common pathogens. Bionanoscience.

[77] Awwad A.M., Amer M.W. (2020). Biosynthesis of copper oxide nanoparticles using ailanthus altissima leaf extract and antibacterial activity. J Taibah Univ Sci.

[78] Chandrasekaran R., Yadav S.A., Sivaperumal S. (2020). Phytosynthesis and characterization of copper oxide nanoparticles using the aqueous extract of beta vulgaris L and evaluation of their antibacterial and anticancer activities. J Cluster Sci.

[79] Chung I., Rahuman A.A., Marimuthu S. (2017). Green synthesis of copper nanoparticles using eclipta prostrata leaves extract and their antioxidant and cytotoxic activities. Exp Ther Med.

[80] Rajamma R., Nair S.G., Khadar F.A., Baskaran B. (2020). IET Nanobiotechnology.

[81] Sulaiman G.M., Tawfeeq A.T., Jaaffer M.D. (2018). Biogenic synthesis of copper oxide nanoparticles using olea europaea leaf extract and evaluation of their toxicity activities: an in vivo and in vitro study. Biotechnol Prog.

[82] Rajeshkumar S., Menon S., Venkat Kumar S. (2019). Antibacterial and antioxidant potential of biosynthesized copper nanoparticles mediated through Cissus arnotiana plant extract. J Photochem Photobiol, B.

[83] Sankar R., Maheswari R., Karthik S., Shivashangari K.S., Ravikumar V. (2014). Anticancer activity of Ficus religiosa engineered copper oxide nanoparticles. Mater Sci Eng C.

[84] Angeline Mary AP., Thaminum Ansari A., Subramanian R. (2019). Sugarcane juice mediated synthesis of copper oxide nanoparticles, characterization and their antibacterial activity. J King Saud Univ Sci.

[85] Selvan S.M., Anand K.V., Govindaraju K. (2018). Green synthesis of copper oxide nanoparticles and mosquito larvicidal activity against dengue, zika and chikungunya causing vector aedes Aegypti. IET Nanobiotechnol.

[86] Narayanan K.B., Sakthivel N., Narayanan K.B., Sakthivel N. (2010). Biological synthesis of metal nanoparticles by microbes. Adv Colloid Interface Sci.

[87] Ahmad A., Senapati S., Khan M.I., Kumar R., Sastry M. (2006). Extra-/Intracellular biosynthesis of gold nanoparticles by an alkalotolerant fungus, *trichothecium* sp. J Biomed Nanotechnol.

[88] Fayaz A.M., Balaji K., Girilal M., Yadav R., Kalaichelvan P.T., Venketesan R. (2010). Biogenic synthesis of silver nanoparticles and their synergistic effect with antibiotics: a study against gram-positive and gram-negative bacteria. Nanomedicine.

[89] Mukherjee P., Ahmad A., Mandal D. (2001). Bioreduction of AuCl4- ions by the fungus, Verticillium sp. and surface trapping of the gold nanoparticles formed. Angew Chem Int Ed.

[90] Shankar S.S., Ahmad A., Pasricha R., Sastry M. (2003). Bioreduction of chloroaurate ions by geranium leaves and its endophytic fungus yields gold nanoparticles of different shapes. J Mater Chem.

[91] Waris A., Din M., Ali A. (2021). A comprehensive review of green synthesis of copper oxide nanoparticles and their diverse biomedical applications. Inorg Chem Commun.

[92] Ghareib M., Abdallah W., Abu Tahon M., Tallima A. (2019). Biosynthesis of copper oxide nanoparticles using the preformed biomass of aspergillus fumigatus and their antibacterial and photocatalytic activities. Dig J Nanomater Biostruct.

[93] Saitawadekar A., Kakde U.B. (2020). Green synthesis of copper nanoparticles using aspergillus flavus. J Crit Rev.

[94] Mousa A.M., Abdel Aziz O.A., Al-Hagar O.E.A., Gizawy M.A., Allan K.F., Attallah M.F. (2020). Biosynthetic new composite material containing CuO nanoparticles produced by Aspergillus terreus for 47Sc separation of cancer theranostics application from irradiated Ca target. Appl Radiat Isot.

[95] Majumder D.R. (2012). Bioremediation: copper nanoparticles from electronic-waste. Int J Eng Sci Technol.

[96] Kovačec E., Regvar M., van Elteren J.T. (2017). Biotransformation of copper oxide nanoparticles by the pathogenic fungus Botrytis cinerea. Chemosphere.

[97] Cristiano José de Andrade B., Maria de Andrade L., Anita Mendes M., Augusto Oller do Nascimento C. (2017).

[98] Cuevas R., Durán N., Diez M.C., Tortella G.R., Rubilar O. (2015). Extracellular biosynthesis of copper and copper oxide nanoparticles by Stereum hirsutum, a native white-rot fungus from chilean forests. J Nanomater.

[99] Consolo V.F., Torres-Nicolini A., Alvarez V.A. (2020). Mycosinthetized Ag, CuO and ZnO nanoparticles from a promising Trichoderma harzianum strain and their antifungal potential against important phytopathogens. Sci Rep.

[100] Li Q., Gadd G.M. (2017). Biosynthesis of copper carbonate nanoparticles by ureolytic fungi. Appl Microbiol Biotechnol.

[101] Singh A V., Patil R., Anand A., Milani P., Gade W.N. (2010). Biological synthesis of copper oxide nano particles using Escherichia coli. Curr Nanosci.

[102] Ghasemi N., Jamali-Sheini F., Zekavati R. (2017). CuO and Ag/CuO nanoparticles: biosynthesis and antibacterial properties. Mater Lett.

[103] Alasvand Zarasvand K., Rai V.R. (2016). Inhibition of a sulfate reducing bacterium, Desulfovibrio marinisediminis GSR3, by biosynthesized copper oxide nanoparticles. 3 Biotech.

[104] Araújo I.M.S., Silva R.R., Pacheco G. (2018). Hydrothermal synthesis of bacterial cellulose–copper oxide nanocomposites and evaluation of their antimicrobial activity. Carbohydr Polym.

[105] Kouhkan M., Ahangar P., Babaganjeh L.A., Allahyari-Devin M. (2019). Biosynthesis of copper oxide nanoparticles using lactobacillus casei subsp. casei and its anticancer and antibacterial activities. Curr Nanosci.

[106] Shantkriti S., Rani P. (2014).

[107] Tiwari M., Jain P., Chandrashekhar Hariharapura R. (2016). Biosynthesis of copper nanoparticles using copper-resistant Bacillus cereus, a soil isolate. Process Biochem.

[108] Ramanathan R., Field M.R., O'Mullane A.P., Smooker P.M., Bhargava S.K., Bansal V. (2013). Aqueous phase synthesis of copper nanoparticles: a link between heavy metal resistance and nanoparticle synthesis ability in bacterial systems. Nanoscale.

[109] Varshney R., Bhadauria S., Gaur M.S., Pasricha R. (2010). Characterization of copper nanoparticles synthesized by a novel microbiological method. J Miner Met Mater Soc.

[110] Bukhari S.I., Hamed M.M., Al-Agamy M.H., Gazwi H.S.S., Radwan H.H., Youssif A.M. (2021). Biosynthesis of copper oxide nanoparticles using streptomyces MHM38 and its biological applications. J Nanomater.

[111] Ghorbani H.R., Mehr F.P., Poor A.K. (2015). Extracellular synthesis of copper nanoparticles using culture supernatants of Salmonella typhimurium. Orient J Chem.

[112] Lv Q., Zhang B., Xing X. (2018). Biosynthesis of copper nanoparticles using Shewanella loihica PV-4 with antibacterial activity: novel approach and mechanisms investigation. J Hazard Mater.

[113] Shah M., Fawcett D., Sharma S., Tripathy S.K., Poinern G.E.J. (2015). Green synthesis of metallic nanoparticles via biological entities. Materials.

[114] Siddiqi K.S., Husen A. (2016). Fabrication of metal and metal oxide nanoparticles by algae and their toxic effects. Nanoscale Res Lett.

[115] Mie R., Samsudin M.W., Din L.B., Ahmad A., Ibrahim N., Adnan S.N.A. (2013). Synthesis of silver nanoparticles with antibacterial activity using the lichen Parmotrema praesorediosum. Int J Nanomed.

[116] Sudha S.S., Rajamanickam K., Rengaramanujam J. (2013). Microalgae mediated synthesis of silver nanoparticles and their antibacterial activity against pathogenic bacteria. Indian J Exp Biol.

[117] Bhattacharya P., Swarnakar S., Ghosh S., Majumdar S., Banerjee S. (2019). Disinfection of drinking water via algae mediated green synthesized copper oxide nanoparticles and its toxicity evaluation. J Environ Chem Eng.

[118] Abboud Y., Saffaj T., Chagraoui A. (2014). Biosynthesis, characterization and antimicrobial activity of copper oxide nanoparticles (CONPs) produced using brown alga extract (Bifurcaria bifurcata). Appl Nanosci.

[119] Arya A., Gupta K., Chundawat T.S., Vaya D. (2018). Biogenic synthesis of copper and silver nanoparticles using green alga botryococcus braunii and its antimicrobial activity. Bioinorgan Chem Appl.

[120] Gu H., Chen X., Chen F., Zhou X., Parsaee Z. (2018). Ultrasound-assisted biosynthesis of CuO-NPs using brown alga Cystoseira trinodis: characterization, photocatalytic AOP, DPPH scavenging and antibacterial investigations. Ultrason Sonochem.

[121] Araya-Castro K., Chao T.C., Durán-Vinet B., Cisternas C., Ciudad G., Rubilar O. (2021). Green synthesis of copper oxide nanoparticles using protein fractions from an aqueous extract of brown algae macrocystis pyrifera. Processes.

[122] Ramaswamy S.V.P., Narendhran S., Sivaraj R. (2016). Potentiating effect of ecofriendly synthesis of copper oxide nanoparticles using brown alga: antimicrobial and anticancer activities. Bull Mater Sci.

[123] Zaitlin B., Watson S.B. (2006). Actinomycetes in relation to taste and odour in drinking water: myths, tenets and truths. Water Res.

[124] Prauser H., Momirova S. (1970). Phage sensitivity, cell wall composition and taxonomy of various thermophilic actinomycetes. Z Allg Mikrobiol.

[125] Manimaran M., Kannabiran K. (2017). Actinomycetes-mediated biogenic synthesis of metal and metal oxide nanoparticles: progress and challenges. Lett Appl Microbiol.

[126] Hassan S.E.D., Fouda A., Radwan A.A. (2019). Endophytic actinomycetes Streptomyces spp mediated biosynthesis of copper oxide nanoparticles as a promising tool for biotechnological applications. J Biol Inorg Chem.

[127] Gebreslassie Y.T., Gebremeskel F.G. (2024). Green and cost-effective biofabrication of copper oxide nanoparticles: exploring antimicrobial and anticancer applications. Biotechnology Reports.

[128] Namakka M., Rahman M.R., Said K.A.M., Abdul Mannan M., Patwary A.M. (2023). A review of nanoparticle synthesis methods, classifications, applications, and characterization. Environ Nanotechnol Monit Manag.

[129] Yuan Y., Lei A. (2020). Is electrosynthesis always green and advantageous compared to traditional methods?. Nat Commun.

[130] Manjunatha C., Ashoka S., Hari Krishna R. (2020). Green Sustainable Process for Chemical and Environmental Engineering and Science: Green Inorganic Synthesis.

[131] Alsamhary K.E. (2023). Moringa oleifera seed based green synthesis of copper nanoparticles: characterization, environmental remediation and antimicrobial activity. Saudi J Biol Sci.

[132] Rajamohan R., Lee Y.R. (2023). Microwave-assisted synthesis of copper oxide nanoparticles by apple peel extract and efficient catalytic reduction on methylene blue and crystal violet. J Mol Struct.

[133] Niculescu A.G., Chircov C., Bîrcă A.C., Grumezescu A.M. (2021).

[134] Yao F., Zhu P., Chen J. (2023). Synthesis of nanoparticles via microfluidic devices and integrated applications. Microchim Acta.

[135] Ahmed M.A., Mohamed A.A. (2024). Advances in ultrasound-assisted synthesis of photocatalysts and sonophotocatalytic processes. A review. iScience.

[136] Jayasimha H.N., Chandrappa K.G., Sanaulla P.F., Dileepkumar V.G. (2024). Green synthesis of CuO nanoparticles: a promising material for photocatalysis and electrochemical sensor. Int Sensor.

[137] Mourdikoudis S., Pallares R.M., Thanh N.T.K. (2018). Characterization techniques for nanoparticles: comparison and complementarity upon studying nanoparticle properties. Nanoscale.

[138] Ibrahim A.M., Munshi G.H., Al-Harbi L.M. (2018). Copper(II) oxide nanocatalyst preparation and characterization: green chemistry route. Bull Natl Res Cent.

[139] Długosz O., Chwastowski J., Banach M. (2020). Hawthorn berries extract for the green synthesis of copper and silver nanoparticles. Chem Paper.

[140] Ismail M.I.M. (2020). Green synthesis and characterizations of copper nanoparticles. Mater Chem Phys.

[141] Ramadhan V.B., Ni'Mah Y.L., Yanuar E., Suprapto S. (2019). AIP Conference Proceedings..

[142] Shi L.B., Tang P.F., Zhang W., Zhao Y.P., Zhang L.C., Zhang H. (2017). Green synthesis of CuO nanoparticles using Cassia auriculata leaf extract and in vitro evaluation of their biocompatibility with rheumatoid arthritis macrophages (RAW 264.7). Trop J Pharmaceut Res.

[143] Rafique M., Shaikh A.J., Rasheed R. (2017). A review on synthesis, characterization and applications of copper nanoparticles using green method. Nano.

[144] Wu C.K., Yin M., O'Brien S., Koberstein J.T. (2006). Quantitative analysis of copper oxide nanoparticle composition and structure by X-ray photoelectron spectroscopy. Chem Mater.

[145] Ebrahim-Saraie H.S., Heidari H., Rezaei V., Mortazavi S.M.J., Motamedifar M. (2018). Promising antibacterial effect of copper oxide nanoparticles against several multidrug resistant uropathogens. Pharmaceut Sci.

[146] Deng Y., Handoko A.D., Du Y., Xi S., Yeo B.S. (2016). In situ Raman spectroscopy of copper and copper oxide surfaces during electrochemical oxygen evolution reaction: identification of CuIII oxides as catalytically active species. ACS Catal.

[147] Naika H.R., Lingaraju K., Manjunath K. (2015). Green synthesis of CuO nanoparticles using Gloriosa superba L. extract and their antibacterial activity. J Taibah Univ Sci.

[148] Nair G.M., Sajini T., Mathew B. (2022). Advanced green approaches for metal and metal oxide nanoparticles synthesis and their environmental applications. Talanta Open.

[149] Jadhav M.S., Kulkarni S., Raikar P., Barretto D.A., Vootla S.K., Raikar U.S. (2018). Green biosynthesis of CuO & Ag-CuO nanoparticles from Malus domestica leaf extract and evaluation of antibacterial, antioxidant and DNA cleavage activities. New J Chem.

[150] Applerot G., Lellouche J., Lipovsky A. (2012). Understanding the antibacterial mechanism of CuO nanoparticles: revealing the route of induced oxidative stress. Small.

[151] Halbus A.F., Horozov T.S., Paunov V.N. (2017). Colloid particle formulations for antimicrobial applications. Adv Colloid Interface Sci.

[152] Shkodenko L., Kassirov I., Koshel E. (2020).

[153] Marambio-Jones C., Hoek E.M.V. (2010). A review of the antibacterial effects of silver nanomaterials and potential implications for human health and the environment. J Nanoparticle Res.

[154] Yugandhar P., Vasavi T., Uma Maheswari Devi P., Savithramma N. (2017). Bioinspired green synthesis of copper oxide nanoparticles from Syzygium alternifolium (Wt.) Walp: characterization and evaluation of its synergistic antimicrobial and anticancer activity. Appl Nanosci.

[155] Shende S., Ingle A.P., Gade A., Rai M. (2015). Green synthesis of copper nanoparticles by Citrus medica Linn. (Idilimbu) juice and its antimicrobial activity. World J Microbiol Biotechnol.

[156] Rajesh K.M., Ajitha B., Reddy Y.A.K., Suneetha Y., Reddy P.S. (2018). Assisted green synthesis of copper nanoparticles using Syzygium aromaticum bud extract: physical, optical and antimicrobial properties. Optik.

[157] El-Batal A.I., El-Sayyad G.S., Mosallam F.M., Fathy R.M. (2020). Penicillium chrysogenum-mediated mycogenic synthesis of copper oxide nanoparticles using gamma rays for in vitro antimicrobial activity against some plant pathogens. J Cluster Sci.

[158] Vishveshvar K., Aravind Krishnan M.V., Haribabu K., Vishnuprasad S. (2018). Green synthesis of copper oxide nanoparticles using ixiro coccinea plant leaves and its characterization. Bionanoscience.

[159] Sharma P., Mehta M., Dhanjal D.S. (2019). Emerging trends in the novel drug delivery approaches for the treatment of lung cancer. Chem Biol Interact.

[160] Bindhu M.R., Umadevi M. (2014). Antibacterial activities of green synthesized gold nanoparticles. Mater Lett.

[161] Society R.S., Hassan E., City S. (2015). Antibacterial activity of synthesized copper oxide nanoparticles using malva sylvestris leaf extract. SMU Medical journal.

[162] Sivaraj R., Rahman P.K.S.M., Rajiv P., Salam H.A., Venckatesh R. (2014). Biogenic copper oxide nanoparticles synthesis using Tabernaemontana divaricate leaf extract and its antibacterial activity against urinary tract pathogen. Spectrochim Acta Mol Biomol Spectrosc.

[163] Sharmila G., Thirumarimurugan M., Sivakumar V.M. (2016). Optical, catalytic and antibacterial properties of phytofabricated CuO nanoparticles using Tecoma castanifolia leaf extract. Optik.

[164] Wu S., Rajeshkumar S., Madasamy M., Mahendran V. (2020). Green synthesis of copper nanoparticles using Cissus vitiginea and its antioxidant and antibacterial activity against urinary tract infection pathogens. Artif Cells, Nanomed Biotechnol.

[165] Velsankar K., Aswin Kumara R.M., Preethi R., Muthulakshmi V., Sudhahar S. (2020). Green synthesis of CuO nanoparticles via Allium sativum extract and its characterizations on antimicrobial, antioxidant, antilarvicidal activities. J Environ Chem Eng.

[166] Gopinath V., Priyadarshini S., Al-Maleki A.R. (2016). In vitro toxicity, apoptosis and antimicrobial effects of phyto-mediated copper oxide nanoparticles. RSC Adv.

[167] Zhao H., Su H., Ahmeda A. (2022). Biosynthesis of copper nanoparticles using Allium eriophyllum Boiss leaf aqueous extract; characterization and analysis of their antimicrobial and cutaneous wound-healing potentials. Appl Organomet Chem.

[168] Sackey J., Nwanya A.C., Bashir A.K.H. (2020). Electrochemical properties of Euphorbia pulcherrima mediated copper oxide nanoparticles. Mater Chem Phys.

[169] Tshireletso P., Ateba C.N., Fayemi O.E. (2021). Spectroscopic and antibacterial properties of CuONPs from orange, lemon and tangerine peel extracts: potential for combating bacterial resistance. Molecules.

[170] Hemmati S., Ahmeda A., Salehabadi Y., Zangeneh A., Zangeneh M.M. (2020). Synthesis, characterization, and evaluation of cytotoxicity, antioxidant, antifungal, antibacterial, and cutaneous wound healing effects of copper nanoparticles using the aqueous extract of Strawberry fruit and L-Ascorbic acid. Polyhedron.

[171] Nagaraj E., Karuppannan K., Shanmugam P., Venugopal S. (2019). Exploration of bio-synthesized copper oxide nanoparticles using Pterolobium hexapetalum leaf extract by photocatalytic activity and biological evaluations. J Cluster Sci.

[172] Cui W.Y., Yoo H.J., Li Y.G., Baek C., Min J. (2021). Electrospun nanofibers embedded with copper oxide nanoparticles to improve antiviral function. J Nanosci Nanotechnol.

[173] Tortella G., Rubilar O., Fincheira P. (2021). Bactericidal and virucidal activities of biogenic metal-based nanoparticles: advances and perspectives. Antibiotics.

[174] Merkl P., Long S., McInerney G.M., Sotiriou G.A. (2021). Antiviral activity of silver, copper oxide and zinc oxide nanoparticle coatings against sars-cov-2. Nanomaterials.

[175] do Carmo Neto J.R., Guerra R.O., Machado J.R., Silva A.C.A., da Silva M.V. (2021). Antiprotozoal and anthelmintic activity of zinc oxide nanoparticles. Curr Med Chem.

[176] Franco A.M.R., Grafova I., Soares F.V. (2016). Nanoscaled hydrated antimony (V) oxide as a new approach to first-line antileishmanial drugs. Int J Nanomed.

[177] Pansambal S., Deshmukh K., Savale A. (2017). Phytosynthesis and biological activities of fluorescent CuO nanoparticles using acanthospermum hispidum L. extract. Journal of Nanostructures.

[178] Faisal S., Al-Radadi N.S., Jan HAbdullah (2021). Curcuma longa mediated synthesis of copper oxide, nickel oxide and Cu-Ni bimetallic hybrid nanoparticles: characterization and evaluation for antimicrobial, anti-parasitic and cytotoxic potentials. Coatings.

[179] Powar N.S., Patel V.J., Pagare P.K., Pandav R.S. (2019). Chemical methodologies Cu nanoparticle: synthesis, characterization and application graphical abstract. Chemical Methodologies.

[180] Diaz-Droguett D.E., Espinoza R., Fuenzalida V.M. (2011). Copper nanoparticles grown under hydrogen: study of the surface oxide. Appl Surf Sci.

[181] Liu H., Zheng S.M., Xiong H.F., Alwahibi M.S., Niu X. (2020). Biosynthesis of copperoxide nanoparticles using Abies spectabilis plant extract and analyzing its antinociceptive and anti-inflammatory potency in various mice models. Arab J Chem.

[182] Zangeneh M.M., Ghaneialvar H., Akbaribazm M. (2019). Novel synthesis of Falcaria vulgaris leaf extract conjugated copper nanoparticles with potent cytotoxicity, antioxidant, antifungal, antibacterial, and cutaneous wound healing activities under in vitro and in vivo condition. J Photochem Photobiol, B.

[183] Tao X., Wan X., Wu D., Song E., Song Y. (2021). A tandem activation of NLRP3 inflammasome induced by copper oxide nanoparticles and dissolved copper ion in J774A.1 macrophage. J Hazard Mater.

[184] Ruiz P., Katsumiti A., Nieto J.A. (2015). Short-term effects on antioxidant enzymes and long-term genotoxic and carcinogenic potential of CuO nanoparticles compared to bulk CuO and ionic copper in mussels Mytilus galloprovincialis. Mar Environ Res.

[185] Canli E.G., Ila H.B., Canli M. (2019). Response of the antioxidant enzymes of rats following oral administration of metal-oxide nanoparticles (Al 2 O 3 , CuO, TiO 2). Environ Sci Pollut Control Ser.

[186] Vinothkanna A., Mathivanan K., Ananth S., Ma Y., Sekar S. (2023). Biosynthesis of copper oxide nanoparticles using Rubia cordifolia bark extract: characterization, antibacterial, antioxidant, larvicidal and photocatalytic activities. Environ Sci Pollut Control Ser.

[187] Kumar B., Smita K., Cumbal L., Debut A., Angulo Y. (2017). Biofabrication of copper oxide nanoparticles using Andean blackberry (Rubus glaucus Benth.) fruit and leaf. J Saudi Chem Soc.

[188] Asghar M., Sajjad A., Hanif S., Ali J.S., Zeeshan M., Zia M. (2022). Comparative analysis of synthesis, characterization, antimicrobial, antioxidant, and enzyme inhibition potential of roses petal based synthesized copper oxide nanoparticles. Mater Chem Phys.

[189] Singh J., Kumar V., Kim K.H., Rawat M. (2019). Biogenic synthesis of copper oxide nanoparticles using plant extract and its prodigious potential for photocatalytic degradation of dyes. Environ Res.

[190] Saravanakumar K., Shanmugam S., Varukattu N.B., MubarakAli D., Kathiresan K., Wang M.H. (2019). Biosynthesis and characterization of copper oxide nanoparticles from indigenous fungi and its effect of photothermolysis on human lung carcinoma. J Photochem Photobiol, B.

[191] Abd Kelkawi A.H., Kajani A.A., Bordbar A.K. (2017). Green synthesis of silver nanoparticles using Mentha pulegium and investigation of their antibacterial, antifungal and anticancer activity. IET Nanobiotechnol.

[192] Sok S.P.M., Arshad N.M., Azmi M.N., Awang K., Ozpolat B., Nagoor N.H. (2017). The apoptotic effect of 1′S-1′-Acetoxychavicol Acetate (ACA) enhanced by inhibition of non-canonical autophagy in human non-small cell lung cancer cells. PLoS One.

[193] Zakaria N., Mahdzir M.A., Yusoff M., Arshad N.M., Awang K., Nagoor N.H. (2018). Cytotoxic effects of pinnatane A extracted from walsura pinnata (meliaceae) on human liver cancer cells. Molecules.

[194] Riss T.L., Niles A.L., Minor L. (2004).

[195] Akintelu S.A., Folorunso A.S., Folorunso F.A., Oyebamiji A.K. (2020). Green synthesis of copper oxide nanoparticles for biomedical application and environmental remediation. Heliyon.

[196] Bharathi D., Ranjithkumar R., Chandarshekar B., Bhuvaneshwari V. (2019). Bio-inspired synthesis of chitosan/copper oxide nanocomposite using rutin and their anti-proliferative activity in human lung cancer cells. Int J Biol Macromol.

[197] Harishchandra B.D., Pappuswamy M., Pu A. (2020). Copper nanoparticles: a review on synthesis, characterization and applications. J Asian Pac Cancer Biol.

[198] Siddiqui M.A., Alhadlaq H.A., Ahmad J., Al-Khedhairy A.A., Musarrat J., Ahamed M. (2013). Copper oxide nanoparticles induced mitochondria mediated apoptosis in human hepatocarcinoma cells. PLoS One.

[199] Barabadi H., Vahidi H., Damavandi Kamali K., Rashedi M., Saravanan M. (2020). Antineoplastic biogenic silver nanomaterials to combat cervical cancer: a novel approach in cancer therapeutics. J Cluster Sci.

[200] Khatua A., Prasad A., Priyadarshini E. (2020). Emerging antineoplastic plant-based gold nanoparticle synthesis: a mechanistic exploration of their anticancer activity toward cervical cancer cells. J Cluster Sci.

[201] Barabadi H., Vahidi H., Mahjoub M.A. (2020). Emerging antineoplastic gold nanomaterials for cervical cancer therapeutics: a systematic review. J Cluster Sci.

[202] Barabadi H., Vahidi H., Damavandi Kamali K. (2020). Emerging theranostic gold nanomaterials to combat lung cancer: a systematic review. J Cluster Sci.

[203] Raj Preeth D., Shairam M., Suganya N. (2019). Green synthesis of copper oxide nanoparticles using sinapic acid: an underpinning step towards antiangiogenic therapy for breast cancer. J Biol Inorg Chem.

[204] Mendhulkar V.D., Anu Y. (2017). Anticancer activity of Camellia Sinensis mediated copper nanoparticles against HT-29, MCF-7, and MOLT-4 human cancer cell lines. Asian J Pharmaceut Clin Res.

[205] Malaikozhundan B., Vinodhini J., Kalanjiam M.A.R. (2020). High synergistic antibacterial, antibiofilm, antidiabetic and antimetabolic activity of Withania somnifera leaf extract-assisted zinc oxide nanoparticle. Bioproc Biosyst Eng.

[206] Faisal S., Jan H., Abdullah Alam I. (2022). In vivo analgesic, anti-inflammatory, and anti-diabetic screening of Bacopa monnieri-synthesized copper oxide nanoparticles. ACS Omega.

[207] Arumai Selvan DS., Kumar R.S., Murugesan S., Shobana S., Rahiman A.K. (2022). Antidiabetic activity of phytosynthesized Ag/CuO nanocomposites using Murraya koenigii and Zingiber officinale extracts. J Drug Deliv Sci Technol.

[208] Xu V.W., Nizami M.Z.I., Yin I.X., Yu O.Y., Lung C.Y.K., Chu C.H. (2022). Application of copper nanoparticles in dentistry. Nanomaterials.

[209] Grass G., Rensing C., Solioz M. (2011). Metallic copper as an antimicrobial surface. Appl Environ Microbiol.

[210] Liu H., Tang Y., Zhang S. (2022). Anti-infection mechanism of a novel dental implant made of titanium-copper (TiCu) alloy and its mechanism associated with oral microbiology. Bioact Mater.

[211] Koohkan R., Hooshmand T., Tahriri M., Mohebbi-Kalhori D. (2018). Synthesis, characterization and in vitro bioactivity of mesoporous copper silicate bioactive glasses. Ceram Int.

[212] Astasov-Frauenhoffer M., Koegel S., Waltimo T. (2019). Antimicrobial efficacy of copper-doped titanium surfaces for dental implants. J Mater Sci Mater Med.

[213] Ali Thamer N., Tareq Barakat N. (2019). Journal of Physics: Conference Series.

[214] Rubilar O., Rai M., Tortella G., Diez M.C., Seabra A.B., Durán N. (2013).

[215] Natrayan L., Kaliappan S., Saravanan A. (2023). Recyclability and catalytic characteristics of copper oxide nanoparticles derived from bougainvillea plant flower extract for biomedical application. Green Process Synth.

[216] Mandal S., De S. (2015). Catalytic and fluorescence studies with copper nanoparticles synthesized in polysorbates of varying hydrophobicity. Colloids Surf A Physicochem Eng Asp.

[217] Pakzad K., Alinezhad H., Nasrollahzadeh M. (2019). Green synthesis of Ni@Fe3O4 and CuO nanoparticles using Euphorbia maculata extract as photocatalysts for the degradation of organic pollutants under UV-irradiation. Ceram Int.

[218] Bordbar M., Sharifi-Zarchi Z., Khodadadi B. (2017). Green synthesis of copper oxide nanoparticles/clinoptilolite using Rheum palmatum L. root extract: high catalytic activity for reduction of 4-nitro phenol, rhodamine B, and methylene blue. J Sol Gel Sci Technol.

[219] Asghar M.A., Zahir E., Shahid S.M. (2018). Iron, copper and silver nanoparticles: green synthesis using green and black tea leaves extracts and evaluation of antibacterial, antifungal and aflatoxin B1 adsorption activity. Lebensm Wiss Technol.

[220] Maithreyee M.N., Gowda R. (2015). Influence of nanoparticles in enhancing seed quality of aged seeds. Mysore J Agric Sci.

[221] Nagaonkar D., Shende S., Rai M. (2015). Biosynthesis of copper nanoparticles and its effect on actively dividing cells of mitosis in Allium cepa. Biotechnol Prog.

[222] Nair P.M.G., Chung I.M. (2014). Impact of copper oxide nanoparticles exposure on Arabidopsis thaliana growth, root system development, root lignificaion, and molecular level changes. Environ Sci Pollut Control Ser.

[223] Atha D.H., Wang H., Petersen E.J. (2012). Copper oxide nanoparticle mediated DNA damage in terrestrial plant models. Environ Sci Technol.

[224] Yasmeen F., Raja N.I., Razzaq A., Komatsu S. (2017). Proteomic and physiological analyses of wheat seeds exposed to copper and iron nanoparticles. Biochim Biophys Acta, Proteins Proteomics.

[225] Kasana R.C., Panwar N.R., Kaul R.K., Kumar P. (2017). Biosynthesis and effects of copper nanoparticles on plants. Environ Chem Lett.

[226] Sarkar J., Chakraborty N., Chatterjee A., Bhattacharjee A., Dasgupta D., Acharya K. (2020). Green synthesized copper oxide nanoparticles ameliorate defence and antioxidant enzymes in Lens culinaris. Nanomaterials.

[227] Bilal Tahir M., Nabi G., Rafique M., Khalid N.R. (2018). Role of fullerene to improve the WO3 performance for photocatalytic applications and hydrogen evolution. Int J Energy Res.

[228] Tahir M.B., Sagir M., Abas N. (2019). Enhanced photocatalytic performance of CdO-WO3 composite for hydrogen production. Int J Hydrogen Energy.

[229] Kerour A., Boudjadar S., Bourzami R., Allouche B. (2018). Eco-friendly synthesis of cuprous oxide (Cu2O) nanoparticles and improvement of their solar photocatalytic activities. J Solid State Chem.

[230] Pérez García P.M., Ibáñez-Calero S.L. (2017). Degradation of synthetic organic dyes in solution by ferrate – hypochlorite or calcium hypochlorite. INVESTIGACION & DESARROLLO.

[231] Nasrollahzadeh M., Sajadi S.M., Rostami-Vartooni A., Hussin S.M. (2016). Green synthesis of CuO nanoparticles using aqueous extract of Thymus vulgaris L. leaves and their catalytic performance for N-arylation of indoles and amines. J Colloid Interface Sci.

[232] Kandhola G., Park S., Lim J.W. (2023). Nanomaterial-based scaffolds for tissue engineering applications: a review on graphene, carbon nanotubes and nanocellulose. J Regen Med Tissue Eng.

[233] Hou Y., Wang W., Bartolo P. (2022). Investigation of polycaprolactone for bone tissue engineering scaffolds: in vitro degradation and biological studies. Mater Des.

[234] Wei M., Li S., Le W. (2017). Nanomaterials modulate stem cell differentiation: biological interaction and underlying mechanisms. J Nanobiotechnol.

[235] Sparks H.D., Mandla S., Vizely K., Rosin N., Radisic M., Biernaskie J. (2022). Application of an instructive hydrogel accelerates re-epithelialization of xenografted human skin wounds. Sci Rep.

[236] Galdiero S., Falanga A., Cantisani M., Ingle A., Galdiero M., Rai M. (2014).

[237] Kirkegaard M.L., Kines P., Jeschke K.C., Jensen K.A. (2020). Risk perceptions and safety cultures in the handling of nanomaterials in academia and industry. Ann Work Expo Health.

[238] Krug H.F. (2022). Collection of controlled nanosafety data—the CoCoN-database, a tool to assess nanomaterial hazard. Nanomaterials.

[239] Solano R., Patiño-Ruiz D., Tejeda-Benitez L., Herrera A. (2021). Metal- and metal/oxide-based engineered nanoparticles and nanostructures: a review on the applications, nanotoxicological effects, and risk control strategies. Environ Sci Pollut Control Ser.

[240] Wang Z., Li N., Zhao J., White J.C., Qu P., Xing B. (2012). CuO nanoparticle interaction with human epithelial cells: cellular uptake, location, export, and genotoxicity. Chem Res Toxicol.

[241] Oberdörster G., Elder A., Rinderknecht A. (2009). Nanoparticles and the brain: cause for concern?. J Nanosci Nanotechnol.

[242] Scherer F., Anton M., Schillinger U. (2002). Magnetofection: enhancing and targeting gene delivery by magnetic force in vitro and in vivo. Gene Ther.

[243] Büttner J., Schneider T., Westermann M., Glei M. (2022). Artificial digestion of polydisperse copper oxide nanoparticles: investigation of effects on the human in vitro intestinal Co-culture model caco-2/HT29-MTX. Toxics.

[244] Cao Y., Dhahad H.A., El-Shorbagy M.A. (2021). Green synthesis of bimetallic ZnO–CuO nanoparticles and their cytotoxicity properties. Sci Rep.

[245] Farshori N.N., Siddiqui M.A., Al-Oqail M.M. (2022). Copper oxide nanoparticles exhibit cell death through oxidative stress responses in human airway epithelial cells: a mechanistic study. Biol Trace Elem Res.

[246] Amaregouda Y., Kamanna K. (2022). Physico-chemical, in-vitro cytotoxicity and antimicrobial evaluation of L-valine functionalised CuO NPs on polyvinyl alcohol and blended carboxymethyl cellulose films. Indian Chem Eng.

[247] Naz S., Hanif S., Ali H., Ali J.S., Zia M. (2021). Synthesis, characterization, in vitro and in vivo toxicity of CuO nanoparticles fabricated through Rhus punjabensis leaf extract. Bionanoscience.

[248] Piret J.P., Jacques D., Audinot J.N. (2012). Copper(ii) oxide nanoparticles penetrate into HepG2 cells, exert cytotoxicity via oxidative stress and induce pro-inflammatory response. Nanoscale.

[249] Wang Y., Guo C.X., Liu J., Chen T., Yang H., Li C.M. (2011). CeO2 nanoparticles/graphene nanocomposite-based high performance supercapacitor. Dalton Trans.

[250] Bushueva T., Minigalieva I., Panov V. (2019). More data on in vitro assessment of comparative and combined toxicity of metal oxide nanoparticles. Food Chem Toxicol.

[251] Fahmy B., Cormier S.A. (2009). Copper oxide nanoparticles induce oxidative stress and cytotoxicity in airway epithelial cells. Toxicol Vitro.

[252] Costa P.M., Gosens I., Williams A. (2018). Transcriptional profiling reveals gene expression changes associated with inflammation and cell proliferation following short-term inhalation exposure to copper oxide nanoparticles. J Appl Toxicol.

[253] Minigalieva I.A., Katsnelson B.A., Panov V.G. (2017). In vivo toxicity of copper oxide, lead oxide and zinc oxide nanoparticles acting in different combinations and its attenuation with a complex of innocuous bio-protectors. Toxicology.

[254] Ali J.S., Mannan A., Nasrullah M., Ishtiaq H., Naz S., Zia M. (2020). Antimicrobial, antioxidative, and cytotoxic properties of Monotheca buxifolia assisted synthesized metal and metal oxide nanoparticles. Inorg Metal-Org Nano-Metal Chem.

[255] Ahmad A., Rasheed N., Banu N., Palit G. (2010). Alterations in monoamine levels and oxidative systems in frontal cortex, striatum, and hippocampus of the rat brain during chronic unpredictable stress. Stress.

[256] Prabhu B.M., Ali S.F., Murdock R.C., Hussain S.M., Srivatsan M. (2010). Copper nanoparticles exert size and concentration dependent toxicity on somatosensory neurons of rat. Nanotoxicology.

[257] Karlsson H.L., Gustafsson J., Cronholm P., Möller L. (2009). Size-dependent toxicity of metal oxide particles-A comparison between nano- and micrometer size. Toxicol Lett.

[258] Tulinska J., Mikusova M.L., Liskova A. (2022). Copper oxide nanoparticles stimulate the immune response and decrease antioxidant defense in mice after six-week inhalation. Front Immunol.

[259] Ouni S., Askri D., Jeljeli M., Abdelmalek H., Sakly M., Amara S. (2020). Toxicity and effects of copper oxide nanoparticles on cognitive performances in rats. Arch Environ Occup Health.

[260] Feidantsis K., Kalogiannis S., Marinoni A. (2020). Toxicity assessment and comparison of the land snail's Cornu aspersum responses against CuO nanoparticles and ZnO nanoparticles. Comparative Biochemistry and Physiology Part - C: toxicology and. Pharmacology.

[261] Lee G., Kim B.S. (2014). Biological reduction of graphene oxide using plant leaf extracts. Biotechnol Prog.

[262] Gosens I., Costa P.M., Olsson M. (2021). Pulmonary toxicity and gene expression changes after short-term inhalation exposure to surface-modified copper oxide nanoparticles. NanoImpact.

[263] Hassanen E.I., Ibrahim M.A., Hassan A.M., Mehanna S., Aljuaydi S.H., Issa M.Y. (2021). Neuropathological and cognitive effects induced by CuO-NPs in rats and trials for prevention using pomegranate juice. Neurochem Res.

[264] Naz S., Nasir B., Ali H., Zia M. (2021). Comparative toxicity of green and chemically synthesized CuO NPs during pregnancy and lactation in rats and offspring: Part I -hepatotoxicity. Chemosphere.

[265] Areecheewakul S., Adamcakova-Dodd A., Haque E. (2022). Time course of pulmonary inflammation and trace element biodistribution during and after sub-acute inhalation exposure to copper oxide nanoparticles in a murine model. Part Fibre Toxicol.

[266] Meghana S., Kabra P., Chakraborty S., Padmavathy N. (2015). Understanding the pathway of antibacterial activity of copper oxide nanoparticles. RSC Adv.

[267] Waris A., Din M., Ali A. (2021). A comprehensive review of green synthesis of copper oxide nanoparticles and their diverse biomedical applications. Inorg Chem Commun.

[268] Jahangirian H., Lemraski E.G., Webster T.J., Rafiee-Moghaddam R., Abdollahi Y. (2017). A review of drug delivery systems based on nanotechnology and green chemistry: green nanomedicine. Int J Nanomed.

[269] Lam P.L., Wong W.Y., Bian Z., Chui C.H., Gambari R. (2017). Recent advances in green nanoparticulate systems for drug delivery: efficient delivery and safety concern. Nanomedicine.

[270] Navya P.N., Kaphle A., Srinivas S.P., Bhargava S.K., Rotello V.M., Daima H.K. (2019). Current trends and challenges in cancer management and therapy using designer nanomaterials. Nano Converg.

[271] Jose Varghese R., Zikalala N., Sakho E.H.M., Oluwafemi O.S. (2020). Colloidal Metal Oxide Nanoparticles.

[272] Fierascu I., Fierascu I.C., Brazdis R.I., Baroi A.M., Fistos T., Fierascu R.C. (2020).

[273] Lan T.D., Huong D.T.T., Trang C.T.T. (2012). Proceedings of the 12th International Coral Reef Symposium.

